# Gut microbial metabolism of Flutamide attenuates its therapeutic efficacy against prostate cancer

**DOI:** 10.1080/19490976.2026.2682803

**Published:** 2026-06-07

**Authors:** Shujing Li, Haiying Ding, Jiaqi Wang, Lingjie Yuan, Ying Zhou, Weiben Xu, Hang Yin, Mengqian Ye, Yuning Sha, Fangyin Li, Yousheng Liu, Zhengqin Zhu, Lulu Song, Xiangyu Jin, Liefeng Ma, Zhajun Zhan, Libin Pan, Luo Fang

**Affiliations:** a Department of Pharmacy, Zhejiang Cancer Hospital, Hangzhou Institute of Medicine (HIM), Chinese Academy of Sciences, Hangzhou, China; b College of Pharmaceutical Science, Zhejiang University of Technology, Hangzhou, China; c Institute of Pharmacology and Toxicology, Zhejiang Province Key Laboratory of Anti-Cancer Drug Research, College of Pharmaceutical Sciences, Zhejiang University, Hangzhou, China; d Department of Urology, Zhejiang Cancer Hospital, Hangzhou Institute of Medicine (HIM), Chinese Academy of Sciences, Hangzhou, China; e Hangzhou Institute of Medicine (HIM), Chinese Academy of Sciences, Hangzhou, China; f Postgraduate Training Base Alliance of Wenzhou Medical University (Zhejiang Cancer Hospital), Hangzhou, China

**Keywords:** Gut microbiota, flutamide, prostate cancer, nitroreductase

## Abstract

Endocrine drugs serve as the cornerstone of prostate cancer treatment. Flutamide, a representative first-generation antiandrogen, has been relegated to the treatment of recurrent prostate cancer due to novel drug development and therapeutic resistance. Our study shows the gut microbiota contributes to this resistance. Specifically, gut bacteria metabolize Flutamide into FLU-6 (a nitroreduction product) and FLU-9 (an acetylation product), involving species like *Escherichia coli*. Gene knockout revealed *E. coli nfsA* and *nfsB* as essential for Flutamide nitroreduction, while heterologous expression confirmed acetyltransferases mediate the production of acetylated metabolites. In the antibiotic-treated mouse model, antibiotic intervention significantly reduced microbial metabolites of Flutamide. In addition, FLU-6 was further metabolized by the host into FLU-5. Synthesized FLU-6, FLU-9, and FLU-5 showed no anticancer activity in prostate cancer cell lines. In a xenograft model, oral administration of *E. coli* diminished Flutamide's efficacy by altering its metabolic profile. Clinical sample analysis revealed substantial interpatient variability, and patients could be categorized into subgroups with high or low metabolic capability. These findings provide new insights into personalized prostate cancer therapy, highlight the role of the gut microbiota in Flutamide response and suggest a strategy for optimizing antiandrogen treatments.

## Graphical abstract

**Figure:**
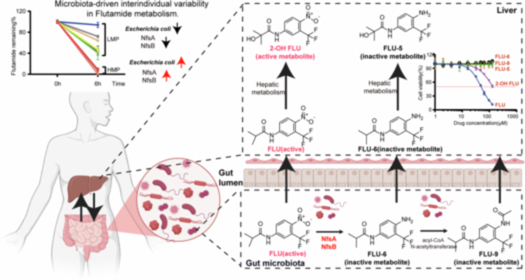

## Introduction

Prostate cancer is the second most prevalent cancer globally and the fifth leading cause of cancer-related mortality among men.^1^ As the cornerstone treatment for advanced prostate cancer, androgen deprivation therapy (ADT) has been used for over 80 y and applies to nearly all phases of prostate cancer.^2^ ^,^ ^3^ ADT inhibits tumor growth by reducing androgen level or blocking androgen binding to the androgen receptor (AR) by surgical intervention (orchidectomy) or agents, including gonadotropin-releasing hormone (GnRH) agonists, androgen synthesis inhibitors, or AR antagonists such as Flutamide (FLU).^4^

Flutamide, a first-generation nonsteroidal antiandrogen, competitively blocks AR activation.^5^ Although once widely used, it has been largely replaced by second-generation agents (e.g., enzalutamide and abiraterone) or by bicalutamide. Flutamide is now recommended for specific clinical scenarios, such as second-line treatment of nonmetastatic castration-resistant prostate cancer in resource-limited settings.^6^ This shift is due to Flutamide's limited efficacy, risk of hepatotoxicity, and the development of resistance.^7^ Therefore, resistance to Flutamide^8^ ^,^ ^9^ and significant interindividual variability^10-12^ in treatment response remain clinically relevant challenges. These characteristics, combined with its well-characterized structure and metabolism, make Flutamide a valuable model compound for investigating mechanisms of antiandrogen resistance and drug–microbiota interactions. Beyond Flutamide, clinical resistance and interindividual differences represent a shared challenge across androgen deprivation therapy (ADT) agents.^13^

The underlying mechanism of ADT resistance is multifaceted, involving AR signaling alterations, tumor microenvironment, hormones.^14^ In parallel, increasing attention has been given to the role of the gut microbiota in oncology, including prostate cancer, through effects on immune regulation and hormonal metabolism.^15–17^ Recently, systematic reviews highlight that gut microbiota remarkably influences the response to ADT in prostate cancer via immune modulation and by directly subverting ADT-related pathways.^16^ For instance, the androgen synthesis inhibitor abiraterone acetate restructured microbial communities in prostate cancer patients. It was metabolized by bacteria, leading to impaired treatment response.^18^ ^,^ ^19^ However, the impact of gut microbes on the disposition and therapeutic effect of Flutamide remains unknown.

Gut microbes have been investigated for their key roles in individual drug disposition and clinical response, particularly direct modulation of drug bioavailability.^20^ For instance, bacteria can mediate the acetylation of 5-aminosalicylic acid, thereby reducing its therapeutic activity in inflammatory bowel disease,^21^ inactivate digoxin in congestive heart failure via its reduction to dihydrodigoxin,^22^ ^,^ ^23^ and cause hepatotoxicity from Brivudine by metabolizing it to bromovinyluracil.^24^ Given the marked heterogeneity in gut microbial composition and functional profiles across individuals, this heterogeneity is anticipated to lead to significant interindividual differences in drug disposition and therapeutic efficacy.^25-27^

The aim of the present study is to explore the role of gut microbiota in modulating Flutamide metabolism and antitumor efficacy. Specifically, we investigated whether intestinal bacteria, particularly *Escherichia coli*, metabolize Flutamide into inactive derivatives, and further assessed how microbial metabolites of Flutamide influence its efficacy. We also examined individual variability in microbial metabolism using clinical samples. These findings provide a theoretical foundation for personalized therapy and elucidating resistance to Flutamide.

## Methods

### Chemicals and reagents

The following compounds were purchased from Shanghai Macklin Biochemical Co. Ltd. (Shanghai, China): Flutamide, hydroxyflutamide (2-OH FLU), menadione, sodium carboxymethylcellulose, cefadroxil, 2-iodosobenzoic acid, erythromycin, and oxytetracycline. Acetyl chloride was obtained from Hangzhou Shuangmu Chemical Co. Ltd. (Hangzhou, China); and B_2_(OH)_4_ and 4,4'-bipyridine were purchased from Shanghai Bide Pharmatech Co. Ltd. (Shanghai, China). *N*, *N*-Dimethylformamide was obtained from Sinopharm Chemical Reagent Co. Ltd. (Beijing, China). MRS and LB media were obtained from Solarbio Biotechnology Co. Ltd. (Beijing, China). Modified Gifu anaerobic medium(mGAM) and MPYG medium were bought from Qingdao Hope Biotechnology Co. Ltd. (Qingdao, China). FLU-6 (*N*-[4-Amino-3-(trifluoromethyl)phenyl]-2-methylpropanamide) was bought from Toronto Research Chemicals, Inc. (Toronto, Canada). Chromatography-grade methanol and acetonitrile were obtained from Merck (Darmstadt, Germany).

### Animals

Male Sprague–Dawley (SD) rats (6 weeks old, 180–200 g), male BALB/c nude mice (4 weeks old, 12–14 g), and C57BL/6 mice (8 weeks old, 20–24 g) were obtained from Shanghai SLAC Laboratory Animal Co. Ltd. (Shanghai, China). The animals were maintained in an environment with a temperature of 22–24 °C and humidity of 45% with a 12-h light/dark cycle (lighting time: 08:00–20:00). All animals were housed for 3 d before the commencement of the experiments, during which they were provided with a standard diet and free access to water. All experimental procedures were conducted in strict accordance with the institutional guidelines for the care and use of laboratory animals and were approved by the Ethics Committee of Zhejiang Cancer Hospital (Approval No.: 2024-05-007, 2024-05-010, 2025-04-019,2025-04-020).

### Bacterial strain selection

To ensure diversity and relevance, bacterial strains used in this study were selected based on previously published literature,^28-30^ with a focus on core members of the human gut microbiota. A total of 21 strains representing 15 genera were included, covering phylogenetically and functionally representative taxa commonly found in the human gut. Detailed information on strain sources, cultivation methods, and protocols can be found in Supplementary Table S1.

### Tumor cell lines and cell culture

Prostate cancer cell lines 22Rv1, LNCaP, Myc-CaP, and the normal liver cell line MIHA, were purchased from Zhejiang Meisen Cell Technology Co. Ltd. (Zhejiang, China). 22Rv1 and LNCaP cells were cultured in Roswell Park Memorial Institute (RPMI)-1640 medium (HyClone Laboratories, Logan, UT, USA), whereas Myc-CaP and MIHA cells were cultured in Dulbecco's modified Eagle's medium (DMEM) (HyClone Laboratories). All cell lines were maintained at 37 °C in a humidified incubator with 5% CO_2_ in accordance with standard laboratory practice. The culture media were supplemented with 100 U/mL penicillin, 100 μg/mL streptomycin (Zhejiang Senrui Biotechnology Co. Ltd., Zhejiang, China), and 10% (v/v) fetal bovine serum (Gibco, Australia).

### *Ex vivo* mixed culture metabolism of Flutamide

As previously reported,^31^ ^,^ ^32^ the metabolism of Flutamide by gut microbiota was investigated using an *ex vivo* mixed bacterial culture system. Intestinal contents from 4–8-week-old male SD rats were collected and mixed with mGAM medium at a 1:20 (w/v) ratio to establish a gut bacterial incubation system. After thorough mixing, nitrogen (N_2_) was introduced to create anaerobic conditions, followed by preincubation at 37 °C for 30 min. The final Flutamide concentration was 50 μM. Incubations were conducted in a shaker at 37 °C and 200 rpm for 0, 3, 12, or 24 h. Following the completion of each incubation period, the reaction was quenched by adding an equal volume of ice-cold acetonitrile. A 100 μL aliquot of the sample was extracted with 400 μL of precooled methanol. The extraction mixture was then stored for 30 min at −20 °C. After centrifugation at 20,000 × g for 15 min, the supernatants were transferred into new tube and vacuum dried. The samples were redissolved with 100 μL of 80% methanol and centrifuged, after which 10 μL of the supernatant was injected into an HPLC system coupled with a quadrupole-orbitrap mass spectrometer (Q-Exactive Orbitrap MS) for qualitative metabolite analysis and identification. In addition, the structures of main metabolites were predicted using the SIRIUS^33–35^ software. Detailed procedures and protocols for LC-MS/MS analysis are provided in the **Supplementary Materials**.

### *In Vitro* metabolism of Flutamide by mono-cultures of individual bacterial strains

A total of 21 individual gut bacterial strains were tested in mono-culture for their ability to metabolize Flutamide. Each strain was cultured under appropriate anaerobic or aerobic conditions based on its physiological characteristics (detailed in Supplementary Table S1). Cultures were harvested during the logarithmic growth phase, resuspended with PBS, and adjusted to an OD_600_ of 0.8.

Subsequently, 6 μL of Flutamide stock solution (5 mM in methanol) was added to 594 μL of bacterial suspension, resulting in a final concentration of 50 μM. Cultures were incubated with Flutamide for 0 or 24 h under appropriate conditions. At the end of each incubation, the reaction was quenched by adding an equal volume of ice-cold acetonitrile, and the samples were frozen at −80 °C. After thawing on ice, 200 μL of the sample was mixed with 200 μL of ice-cold acetonitrile, vortexed for 3 min, and incubated at −20 °C for 1 h to facilitate protein precipitation. The mixture was then centrifuged at 12,500 rpm for 10 min at 4 °C. A 10 μL aliquot of the supernatant was analyzed using HPLC coupled with an Orbitrap Exploris^TM^ 120 mass spectrometer to identify Flutamide metabolites. Detailed procedures and protocols for LC-MS/MS analysis are provided in the Supplementary Materials.

### Generation of the *nfsA/nfsB*-deficient strain of *Escherichia coli*

The CRISPR/Cas gene editing system was used to knockout the *nfsA* and *nfsB* genes in *E. coli* ATCC25922. The N20 guide sequences were designed using the CHOPCHOP platform (https://chopchop.cbu.uib.no) to facilitate targeted knockout of the *nfsA* and *nfsB* genes, as described below: The N20 spacer sequence for *nfsB* (and *nfsA*) was designed as follows: 5′-AAACAGGTTTATCTCAACGT-3′ (*nfsA*: 5′-AATAATCCAGAATAAATGGG-3′). Subsequently, primers pTB1-F/pTB1-R (*nfsA*: pTA1-F/pTA1-R) were designed based on the plasmid pTargetF sequence for the amplification of sgRNA containing N20 targeting the *nfsB* gene (*nfsA*). Primers pTB2-F/pTB2-R and pTB3-F/pTB3-R (*nfsA*: pTA2-F/pTA2-R, pTA3-F/pTA3-R) were designed to amplify approximately 500 bp of upstream and downstream sequences of *nfsB* (*nfsA*) in *E. coli* (GenBank: CP037449.1). The DNA fragments derived from the plasmid pTargetF, sgRNA, and homologous arms were cloned to construct the pTargetF-*nfsB* and pTargetF-*nfsA* plasmids (Supplementary Table S2). Subsequently, the pCas plasmid was introduced into *E. coli* via electroporation, and the resulting culture was grown on kanamycin (Kan)-containing plates at 30 °C. The presence of the pCas plasmid in *E. coli* was confirmed through polymerase chain reaction (PCR) analysis.

The transformed *E. coli* was inoculated into LB medium containing Kan and cultured overnight at 30 °C. The subsequent day, the culture was diluted 1:100 in fresh LB, allowed to grow to an OD_600_ of 0.3, and induced with L-arabinose (final concentration 30 mmol/L) at 30 °C to express the Red recombinase and Cas9 protein. Cells were washed with 10% glycerol and stored as competent cells. Subsequently, approximately 1 μg of the pTarget-*nfsB*/pTarget-*nfsA* plasmid solution was introduced into the *E. coli* cells (for *nfsA* knockout, the *E. coliΔnfsB* deficient strain was used). Transformants containing pTarget-nfsB/pTarget-nfsA plasmids were selected on plates containing kanamycin (Kan) and spectinomycin (Spe). Verification was performed by PCR. The knockout strains were cultured in an LB medium containing 1 mM isopropyl-*β*-d-thiogalactoside (IPTG) until no growth was observed on Spe-resistant media, indicating elimination of pTargetF-*nfsB*/pTarget-*nfsA*. The pCas plasmid was eliminated by culturing the knockout strains at 42 °C until no growth was observed on the Kan-resistant media. Subsequently, verification was conducted by PCR and sequencing. The PCR-verified transformant with a deletion of the *nfsB* gene and the cured pCas and pTargetF-*nfsB* plasmids was designated *E. coliΔnfsB*, whereas the transformant with both *nfsB* and *nfsA* gene deletions was designated *E. coliΔnfsBΔnfsA.*

### *In vitro* validation of NfsA- and NfsB-mediated Flutamide metabolism

The *E. coli*Δ*nfsB*, *E. coli*Δ*nfsB*Δ*nfsA*, and WT *E. coli* glycerol stocks were inoculated into LB medium and cultured overnight at 37 °C with shaking. The next day, following the measurement of the OD_600_ of the cultures, centrifugation was performed at 5,000 rpm for 10 min at 4 °C. The supernatant was discarded, and the pellets were resuspended in PBS to an OD_600_ of 0.8. Final Flutamide concentration was 50 µM. The cultures were incubated at 37 °C with shaking at 200  rpm for 0, 3, 6, or 12 h. Following the incubation period, an equal volume of ice-cold acetonitrile was added to quench the reaction. After protein precipitation with acetonitrile, 10 μL of the supernatant was analyzed by LC-MS/MS (Orbitrap Exploris™ 120). Detailed analytical procedures are described in the Supplementary Materials.

### Heterologous expression and biotransformation of acetyltransferase enzymes

Based on literature reports, the acetylation process requires specific culture conditions, particularly the presence of hydrogen as an electron donor and carbon dioxide as a carbon source during bacterial growth,^36^ ^,^ ^37^ which makes the reaction challenging to study. Therefore, the acyl-CoA *N*-acetyltransferase enzyme (UniRef90 ID: C7H1G6) from *Faecalibacterium prausnitzii*, a common anaerobic bacterium in the human gut, was selected for metabolic function validation.^21^

The nucleotide sequence encoding the acyl-CoA *N*-acetyltransferase enzyme was codon-optimized and synthesized for expression in *E. coli.* The gene was then inserted into the expression vector pET28b (+) using the *Nco*I and *Xho*I restriction enzyme sites. The accuracy of the final expression vector was confirmed using restriction digestion and DNA sequencing. The constructed plasmid was transformed into *E. coli* BL21(DE3) competent cells. The *E. coli* BL21(DE3) harboring the constructed plasmid was cultured in lysogeny broth (LB) medium with 50 μg/mL kanamycin at 37 °C, 220 rpm for approximately 12 h, with 0.2 mM IPTG added during shaking to induce protein expression. Meanwhile, the BL21(DE3) strain without the plasmid was cultured as a control using the same conditions.

Both cultures were resuspended in PBS to an OD_600_ of 0.8, and 6 µL of the Flutamide stock solution (final Flutamide concentration of 100 µM) was added to 594 µL of the culture. The cultures were incubated at 37 °C with agitation at 200 rpm for 0, 6, and 24 h. Following the incubation period, an equal volume of ice-cold acetonitrile was added to terminate the reaction, and the samples were stored at −80 °C for subsequent analysis. The analytical approach used was identical to that used for the Flutamide incubation of the knockout strains.

### Pharmacokinetics of Flutamide mediated by the gut microbiota in SD rats

Rats were randomly assigned to three experimental groups (*n* = 3 per group):

Groups 1(Control): Received 0.2% CMC-Na and PBS via oral gavage.

Groups 2(Abx): Treated with antibiotics and orally gavaged with PBS.

Groups 3(Abx + EC): Treated with antibiotics followed by reconstitution with WT *E. coli* ATCC25922.

For microbiota depletion, groups 2 and 3 were administered a triple antibiotic cocktail (oxytetracycline 200 mg/kg, erythromycin 200 mg/kg, cefadroxil 100 mg/kg) via oral gavage once daily for three consecutive days.^38^ After a 2-d washout period, group 3 was gavaged daily with 10^9^ CFU/mL of *E. coli* for 3 d.

On the following day, all animals were administered Flutamide (125 mg/kg) by oral gavage. Blood samples (200 μL) were collected via orbital sinus puncture at 0 min, 10 min, 30 min, 60 min, 6 h, 12 h and 24 h post-dose. Serum was isolated by centrifugation and stored at −80 °C until analysis.

### Microsomal incubation

Each 100 μL incubation solution contained 14.9 mM isocitric acid, 0.035 unit/mL isocitric dehydrogenase, 0.15 M MgCl_2_, liver microsomes (Huizhihe Yuansheng Biotechnology Co., Ltd., Suzhou, China), 0.1 M Tris-HCl buffer (pH 7.4). Substrates (Flutamide and FLU-6) were added to the liver microsomal suspension and the reactions were initiated with the addition of NADP/NADPH (27 mM/12 mM) after preincubation for 5 min. All incubation was performed in triplicate in a 37 °C water bath with shaking. After 1 h of incubation, the reactions were terminated by the addition of three volumes of ice-cold acetonitrile. The mixtures were vortexed and centrifuged at 12,500 rpm for 10 min. A 360 μL aliquot of the resulting supernatant was collected and dried under vacuum. The residue was reconstituted in 90 μL of 50% acetonitrile, vortexed thoroughly, and centrifuged briefly to remove particulates. A 10 μL aliquot of the supernatant was analyzed using HPLC coupled with an Orbitrap Exploris^TM^ 120 mass spectrometer to identify Flutamide metabolites.

### Cytotoxicity in cells

To evaluate the effect of Flutamide and its active metabolite 2-OH FLU, along with other metabolites (FLU-6, FLU-9, and FLU-5), on 22Rv1, LNCaP, and Myc-CaP cells, a CCK-8 assay (Cell Counting Kit-8, GipBio) was used. Briefly, the cells were seeded in 96-well plates at a density of 10,000 cells per well for 22Rv1 and 5,000 cells per well for LNCaP and Myc-CaP, and incubated overnight. The compounds were dissolved in DMSO at various concentrations. The control group comprised untreated cells, whereas the blank group comprised a fresh culture medium without cells. Following a designated incubation period (48 h for LNCaP and Myc-CaP cells and 72 h for 22Rv1 cells), CCK-8 reagent was added to each well at a 1:10 ratio (CCK-8: complete medium). Absorbance was determined at 450 nm using a microplate reader (BioTek, USA), and cell viability was calculated using the following formula:

$$\mathrm{Cell viability}(\%)=\left[\frac{\left(\mathrm{sample OD}450-\mathrm{matrix blank OD}450\right)}{\left(\mathrm{control OD}450-\mathrm{matrix blank OD}450\right)}\right]\times 100$$

### Cytotoxicity in human hepatocytes

To assess the hepatotoxicity of Flutamide and its microbial metabolites (FLU-6, FLU-9, 2-OH FLU, and FLU-5), a CCK-8 assay was performed using the human hepatocyte cell line MIHA. Cells were seeded in 96-well plates at a density of 5,000 cells per well and incubated overnight. Test compounds were dissolved in DMSO and applied at the same concentrations used in the prostate cancer cell assays. Control wells contained untreated cells, and blank wells contained only culture medium without cells. After 48 h of drug treatment, CCK-8 reagent (GipBio) was added at a 1:10 ratio (CCK-8: complete medium) and incubated for 1.5 h. Absorbance at 450 nm was measured using a microplate reader (BioTek, USA).

### *E. coli*-mediated modulation of Flutamide antitumor efficacy

A xenograft tumor model was established by subcutaneously inoculating tumor masses formed by 22Rv1 cells into the right dorsal flank of male BALB/c nude mice (4 weeks old, 12–14 g). The mice were randomly divided into three groups (*n* = 5 per group): a control group (model), Flutamide treatment group (FLU), and *E. coli* ATCC25922 intervention group (FLU_EC). The following treatments were administered:

1) Control group (model): mice were administered an equal volume of vehicle (PBS and 0.2% CMC-Na) daily by oral gavage.
2) Flutamide treatment group (FLU): mice were administered Flutamide at a dose of 700 mg/kg via oral gavage, with a volume of 0.1 ml per 10 g of body weight, daily.
3) The *E. coli* intervention group (FLU_EC): mice were initially orally gavaged with *E. coli* at a concentration of 10^10^ CFU/100 μL daily, followed by the administration of Flutamide at a dosage of 700 mg/kg, with a volume of 0.1 ml/10 g body weight.

The Flutamide dosing regimen was determined based on the results of a preliminary experiment. Initial doses of FLU LD (100 mg/kg) and HD (200 mg/kg) did not elicit significant pharmacological effects. After adjusting the doses to FLU LD (400 mg/kg) and FLU HD (700 mg/kg), it was observed that the 700 mg/kg dose effectively inhibited the growth of 22Rv1 tumors. Accordingly, the Flutamide dose used in this study was 700 mg/kg/d. Throughout the experiment, the body weight and dimensions of the tumors (length and width) were recorded every 2‒3 d, and tumor volumes were calculated to monitor the growth of the tumors in each group.

### Shotgun metagenomic sequencing of fecal samples from prostate cancer xenograft mice

#### DNA isolation and library construction

Total DNA was isolated from the sample using a QIAamp® Fast DNA Stool Mini Kit (Qiagen, Hilden, Germany) following the manufacturer's instructions. DNA concentration and integrity were assessed by a NanoDrop2000 spectrophotometer (Thermo Fisher Scientific, Waltham, MA, USA) and agarose gel electrophoresis, respectively. DNA was fragmented by S220 Focused-ultrasonicators (Covaris, USA) and cleaned up by Agencourt AMPure XP beads (Beckman Coulter Co., USA). Then the libraries were constructed using TruSeq Nano DNA LT Sample Preparation Kit (Illumina, San Diego, CA, USA) according to the manufacturer's instructions. The Metagenome sequencing and analysis were conducted by OE Biotech Co., Ltd. (Shanghai, China).

#### Bioinformatic analysis

The libraries were sequenced on an illumina Novaseq 6000 platform and 150 bp paired-end reads were generated. Sequences in the FastQ file were trimmed and filtered using Trimmomatic (v 0.36).^39^ Metagenome assembly was performed using MEGAHIT(v 1.1.2)^40^ ^,^ ^41^ after getting valid reads. Use gaps inside the scaffold as breakpoint to interrupt the scaffold into new contigs (Scaftig), and these new Scaftigs with length >500 bp were retained. ORF prediction of assembled scaffolds using prodigal (v 2.6.3)^42^ was performed and translated into amino acid sequences. The nonredundant gene sets were built for all predicted genes using CDHIT (v 4.5.7). The clustering parameters were 95% identity and 90% coverage. The longest gene was selected as the representative sequence of each gene set. Clean reads of each sample were aligned against the nonredundant gene set (95% identity) using bowtie2 (v 2.2.9), and the abundant information of the gene in the corresponding sample was counted.

The taxonomy of the species was obtained as a result of the corresponding taxonomy database of the NR Library, and the abundance of the species was calculated using the corresponding abundance of the genes. In order to construct the abundance profile on the corresponding taxonomy level, abundance statistics were performed at each level of domain, kingdom, phylum, class, order, family, genus, and species.

### *In vitro* metabolism of fecal samples from patients with prostate cancer

Fecal samples were obtained from patients with histologically confirmed prostate cancer prior to radical prostatectomy (Ethics Approval No. IRB-2024-755 (IIT)). All patients were recruited at the preoperative stage. Each sample was divided into two aliquots: one stored in a 50 mL tube containing fecal microbiota preservation solution (GemPharmatech LLC, Jiangsu, China) for anaerobic incubation experiments, and the other in a 5 mL tube without preservation solution for shotgun metagenomic analysis. All samples were immediately snap-frozen on dry ice and stored at −80 °C until further use. The baseline characteristics of all patients included in this experiment are summarized in Supplementary Tables S3 and S4.

The experimental methods and details for the *in vitro* metabolism of Flutamide are the same as those described in “*Ex vivo* Mixed Culture Metabolism of Flutamide”, and the sample preparation for mass spectrometry was consistent with that described in “*In Vitro* Metabolism of Flutamide by Mono-Cultures of Individual Bacterial Strains”.

### Statistical analysis methods

Data were analyzed using Prism version 9.5.0 (GraphPad Software, CA, USA). Two-tailed unpaired Student's *t*-test was used to evaluate the significant difference between two groups while one-way ANOVA followed by Tukey's multiple comparisons test was applied for three or more groups. For Figures 3(g) and 5, predefined pairwise comparisons were conducted between the indicated groups (Figure 3: control vs. Abx and Abx vs. Abx + EC; Figure 5: Model vs. FLU and FLU vs. FLU_EC). Two-tailed unpaired t-tests with Holm correction were used for these comparisons. Results were presented as mean ± standard error of the mean (SEM) or mean ± standard deviation (SD), and *p *< 0.05 was considered statistically significant.

The remaining experimental methods in this study, including the Shotgun metagenomic sequencing and analysis of human fecal samples, the chemical synthesis of metabolites, the details of the LC-MS/MS analytical procedures, and the bioinformatics-related analytical methods, are provided in the Supplementary Materials.

## Results

### Identification of gut microbial metabolites of Flutamide and the responsible metabolizing strains

**Figure 1.**
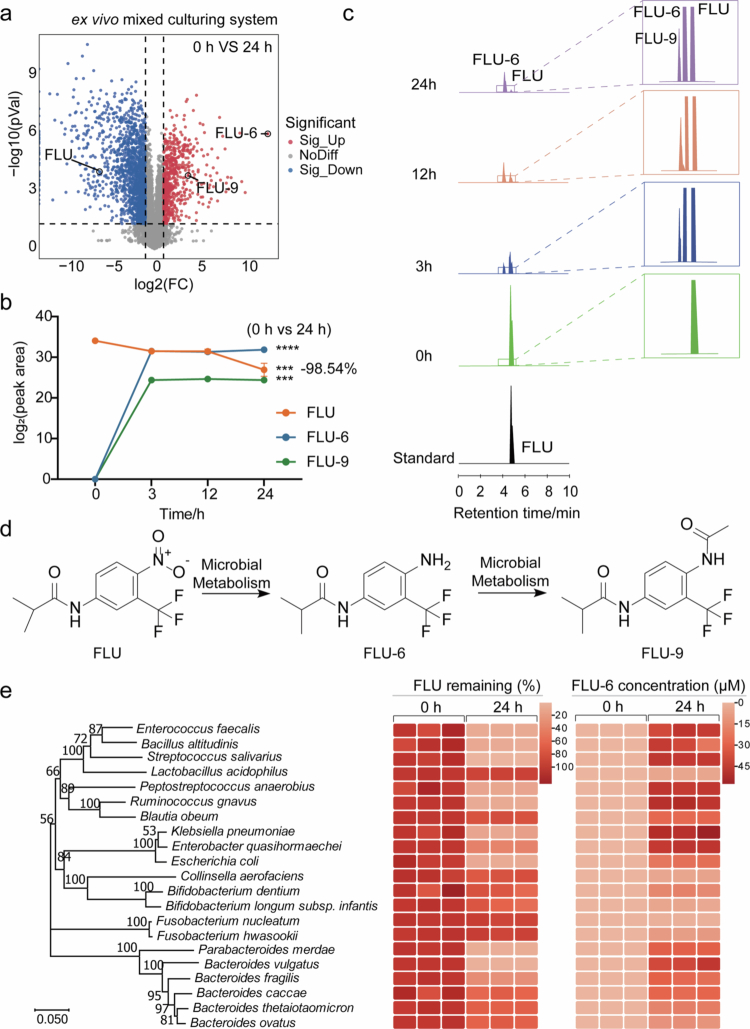
Flutamide is metabolized by the gut microbiota into FLU-6 and FLU-9. (a) Volcano plot of differential metabolites after 0 and 24 h of incubation with Flutamide and gut microbiota. Significantly upregulated and downregulated metabolites (|log_2_ fold change| > 1, *p* < 0.05) are marked in red and blue, respectively. (b) Metabolic curves of Flutamide in the gut microbiota, with the generation of FLU-6 and FLU-9 metabolites at 0, 3, 12, and 24 h. (c) Extracted ion chromatogram (EIC) of FLU, FLU-6, and FLU-9 after incubation with gut microbiota for 0, 3, 12, and 24 h. (d) Molecular structures of FLU and its metabolites (FLU-6 and FLU-9) and the proposed metabolic pathways. (e) Phylogenetic tree of 16S rRNA gene sequences from 21 bacterial strains and heatmap of Flutamide remaining (%) and generated FLU-6 concentration (μM) for each strain. Data are presented as mean ± SEM. ***p < 0.001, ****p < 0.0001 versus 0 h.

To investigate whether Flutamide is metabolized by the gut microbiota, we conducted *ex vivo* anaerobic incubation with a mixed culturing system. Putative metabolites of Flutamide were identified through comparative metabolomic analysis based on mass spectrometry between coincubated samples at 0 h and 24 h. Volcano plot analysis of differential metabolites revealed substantial enrichment of FLU-6 (a previously reported nitroreduction product of Flutamide) and FLU-9 (unknown metabolite) alongside Flutamide depletion after incubation with bacteria (Figure 1a). It was confirmed based on the MS/MS fragment ions and retention times of commercially available standards of Flutamide and FLU-6, and further validated by comparing the Fluorine-19 NMR spectrum profiles before and after metabolism (Supplementary Figure S1(a-c)).

Analysis of MS/MS spectral data using SIRIUS revealed that FLU-6 and FLU-9 shared major fragment ions (*m/z* 245.09, 225.08, 205.08, and 185.07; Supplementary Figure S1d-e, Table S5), indicating a common core structure. Meanwhile, the molecular weight of FLU-9 was 42.01 Da higher than that of FLU-6, suggesting that FLU-9 may be an acetylated derivative of FLU-6. As a novel metabolite and the ninth discovered for Flutamide, it was designated FLU-9. FLU-9 was subsequently obtained and confirmed through chemical synthesis.

Peak area integration using the MSDIAL software confirmed a 98.54% decline in Flutamide after 24 h of coincubation with gut microbiota, with concomitant accumulation of FLU-6 and FLU-9 (Figure 1b). Chromatographic profiles (Figure 1c) displayed a time-dependent enrichment of peaks for FLU-6 (Rt = 4.11 min) and FLU-9 (Rt = 3.91 min), concurrent with the reduction of the Flutamide peak. These data confirm gut microbiota-mediated conversion of Flutamide into FLU-6 and FLU-9. The proposed structures and metabolic relationships are illustrated in Figure 1d.

To further identify which gut microbiota are capable of metabolizing Flutamide, we assessed the Flutamide-metabolizing capacity of 21 core gut bacterial strains from 15 genera^28-30^ ^,^ ^43^ under strain-specific culture conditions. As shown in Figure 1e, these strains exhibited varying metabolic activity of Flutamide, indicating a strain-dependent nature of metabolic activity.

### Structural transformation of Flutamide by gut microbiota via NfsA, NfsB, and acyl-CoA *N*-acetyltransferase

**Figure 2.**
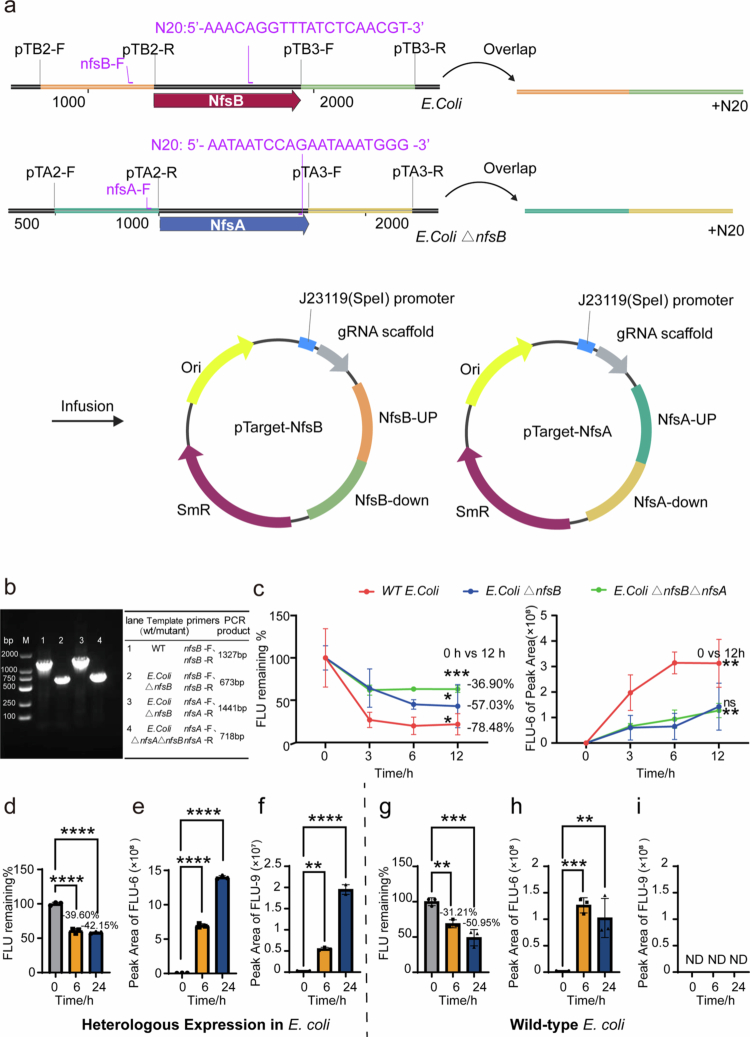
NfsA and NfsB can metabolize Flutamide, and acyl-CoA *N*- acetyltransferase exhibits acetylation activity. (a) Construction of pTarget-*nfsA* and pTarget-*nfsB* plasmids for gene knockout. Spacer sequences targeting *nfsA* or *nfsB* and their homology arms were assembled into the pTarget vector using seamless cloning. These plasmids were used to generate Δ*nfsB* and Δ*nfsA*Δ*nfsB* strains by CRISPR/Cas9. (b) PCR verification of *E. coli* Δ*nfsB* and *E. coli* Δ*nfsA*Δ*nfsB* knockout strains, including agarose gel electrophoresis images of PCR products amplified with specific primer pairs, along with a detailed list of primer sequences (Table S2) and corresponding PCR product sizes. (c) Metabolic curves of Flutamide after incubation with WT *E. coli*, *E. coli* Δ*nfsB*, and *E. coli* Δ*nfsA*Δ*nfsB* at 0, 3, 6, and 12 h, along with the production curve of FLU-6. (d, e, f) Changes in the levels of Flutamide (d), FLU-6 (e), and FLU-9 (f) after co-incubation with *E. coli* heterologously expressing acyl-CoA *N*-acetyltransferase. (g, h, i) Changes in the levels of Flutamide (g), FLU-6 (h), and FLU-9 (i) after coincubation of Flutamide with WT *E. coli*. All percentage values indicate reductions in Flutamide levels after 6 h or 24 h of incubation compared to 0 h. All data are presented as mean ± SD. *p < 0.05, **p < 0.01, ***p < 0.001, and ****p < 0.0001.

Considering the structural transformation of Flutamide into FLU-6 by reduction of a nitro group to an amino group, we hypothesized that the nitroreductases NfsA and NfsB play a pivotal role in this reaction, based on preliminary analysis of the *E. coli* ATCC 25922 genome. To test this hypothesis, we generated *E. coli* ATCC 25922 strains with a single knockout of *nfsB* (Δ*nfsB*) and a double knockout of *nfsA* and *nfsB* (Δ*nfsA*Δ*nfsB*) using CRISPR-Cas9.^36^ The knockout procedure is illustrated in Figure 2a. It was further validated by agarose gel electrophoresis of the PCR products (Figure 2b).

Gradient inhibition of metabolic activity toward Flutamide was observed in the *nfsB* single knockout and Δ*nfsA*Δ*nfsB* double-knockout strain, with a metabolism rate of 57.03% and 36.90%, compared with 78.48% of wild-type *E. coli*. Concomitant decline in FLU-6 production was observed *in vitro* (Figure 2c). It was indicated that NfsA and NfsB are the main contributors to the nitroreduction of Flutamide. To further investigate the role of nitroreductases in Flutamide metabolism, three nitroreductase inhibitors, menadione, dicoumarol, and 2-iodosobenzoic acid, were tested *in vitro*. These compounds inhibited Flutamide metabolism by 55.80%, 36.29%, and 5.42%, respectively. Menadione exhibited the most pronounced inhibition of FLU-6 production (Supplementary Figure S2a). These findings support the key role of nitroreductases in the metabolic conversion of Flutamide.

FLU-9 is postulated as an acetylated metabolite of FLU-6. However, FLU-9 was not detected in the supernatant of individual bacterial strains with Flutamide. To further investigate the potential for acetylation, we heterologously expressed an acyl-CoA *N*-acetyltransferase (UniRef90 ID: C7H1G6) from *Faecalibacterium prausnitzii* in *E. coli*. As illustrated in Figure 2d-f, *E. coli* expressing the heterologous enzyme could proceed with the acetylation reaction following nitroreduction, whereas wild-type *E. coli* did not produce the metabolite FLU-9 (Figure 2g-i). These findings suggested that acyl-CoA *N*-acetyltransferase possesses acetylation activity and plays a pivotal role in Flutamide metabolism.

To investigate strains with potential metabolic capacity of Flutamide, a BLASTp search for the protein sequences of NfsB and NfsA was conducted. A total of 2,684 putative NfsB and 2,616 NfsA homologs were identified. After applying stringent filtering criteria (Query Cover > 80%, E-value < 10^−10^, and identity > 80%), 50 and 64 microbial species were found to possess NfsB and NfsA homologs, respectively (Supplementary Tables S6-7). Phylogenetic analysis demonstrated that NfsB and NfsA were presented in at least 11 and 15 genera, respectively. Except for the genera *Cedecea*, *Buttiauxella*, *Kosakonia*, and *Cronobacter*, which carried only *nfsA*, most strains harbored both the *nfsB* and *nfsA* genes (Supplementary Figure S2(b-c)).

### Gut microbiota-mediated *in vivo* pharmacokinetic study of Flutamide

**Figure 3.**
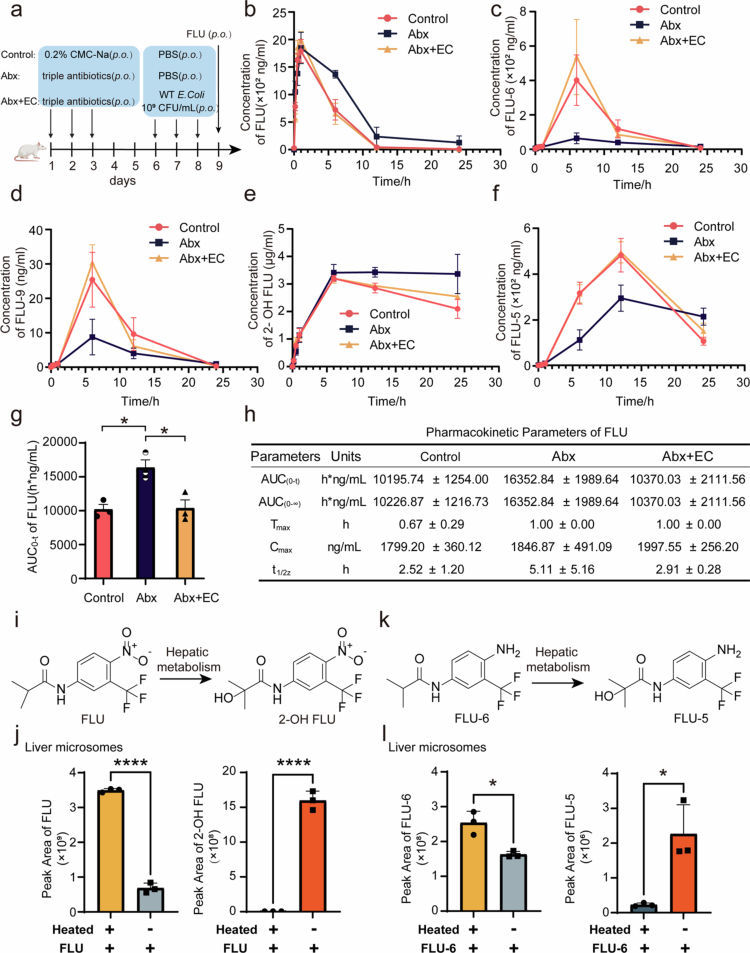
Impact of gut microbiota on the pharmacokinetics of Flutamide in rats. (a) Schematic of the pharmacokinetic experiment following antibiotic treatment and *E. coli* recolonization in rats (*n* = 3 per group). (b-f) Plasma concentration–time curves of FLU (b), FLU-6 (c), FLU-9 (d), 2-OH FLU (e), and FLU-5 (f) in control (Control), antibiotic-treated (Abx), and *E. coli*-recolonized (Abx + EC) rats (*n* = 3 per group). (g) AUC_0-t_ of FLU across the three groups with statistical analysis. (h) Plasma pharmacokinetics parameters of FLU following oral gavage at a dose of 125 mg/kg in Control, Abx, and Abx + EC group rats. (i, j) Chemical structure and proposed hepatic metabolic pathway (i) of FLU to 2-OH FLU, and its incubation with liver microsomes (j). (k, l) Chemical structure and proposed hepatic metabolic pathway (k) of FLU-6 to FLU-5, and its incubation with liver microsomes (l). “Heated” indicates inactivated microsomes. Data in panels (b-g) are presented as mean ± SEM. Data in panels (j,l) are presented as mean ± SD.*p < 0.05, **p < 0.01, ***p < 0.001, and ****p < 0.0001.

Through antibiotic-mediated gut microbiota depletion and subsequent *E. coli* recolonization pharmacokinetic experiments, we evaluated the regulatory role of the gut microbiota in Flutamide metabolism *in vivo* (Figure 3a). The effectiveness of gut microbiota depletion by antibiotics was evaluated in a separate experiment. As shown in Supplementary Figure S3a-d, the fecal DNA content was significantly reduced in the antibiotic-treated group. And independently, 16S rRNA analysis demonstrated substantial alterations in the gut microbial composition. LC-MS/MS analysis of plasma samples revealed a novel microbiota-dependent metabolite, FLU-5, which was initially predicted using SIRIUS software(Supplementary Figure S3e-f) and subsequently synthesized and identified through chemical synthesis. FLU-5 shared characteristic fragment ions with FLU-6 (*m/z* 175.05, 155.04, 135.04, and 115.03; Supplementary Table S8), suggesting it represents a hydroxylation product of FLU-6.

Serum concentration-time curves of Flutamide, FLU-6, FLU-9, 2-OH FLU, and FLU-5 are shown in Figure 3b-f. After effective microbiota depletion by antibiotics, the area under the concentration-time curve (AUC_0-t_) of serum Flutamide significantly increased (10195.74 vs 16352.84 ng/mL·h; *p* = 0.021; Figure 3g-h), after *E. coli* recolonization, the AUC_0-t_ of Flutamide in serum significantly decreased compared to ABX group (16352.84 vs 10370.03 ng/mL·h; *p* = 0.0233; Figure 3g-h). Metabolites derived from gut microbiota, FLU-6 and FLU-9, significantly decreased after antibiotic treatment (Figure 3c-d). In contrast, levels of the hepatic metabolite 2-OH FLU increased (Figure 3e), while levels of FLU-5 (a metabolite of FLU-6) decreased in the antibiotic-treated group (Figure 3f). The levels of the above metabolites were all reversed after *E. coli* recolonization. These findings indicate the gut microbiome's essential role in Flutamide biotransformation.

The origins of 2-OH FLU and FLU-5 warrant further investigation. Liver microsome incubation experiment confirmed that Flutamide undergoes hepatic metabolism to 2-OH FLU, and FLU-6 is further metabolized to FLU-5 by host liver enzymes. The proposed metabolic pathway is illustrated in Figure 3i-l. These results establish 2-OH FLU and FLU-5 as hepatic metabolites.

To investigate the enterohepatic recirculation of Flutamide and its metabolites, we performed bile duct cannulation. Flutamide and its major metabolites, including hepatic metabolites (FLU-5 and 2-OH FLU), and microbiota-derived metabolites (FLU-6 and FLU-9), were detected in bile collected at 0–2 h, 2–4 h, and 4–6 h postadministration, confirming biliary excretion (Supplementary Figure S4a-e). All metabolites were also detected in fecal samples, suggesting the possibility of intestinal reabsorption and metabolism (Supplementary Figure S4f).

### FLU-6, FLU-9, and FLU-5 with diminished anti-prostate cancer activity

**Figure 4.**
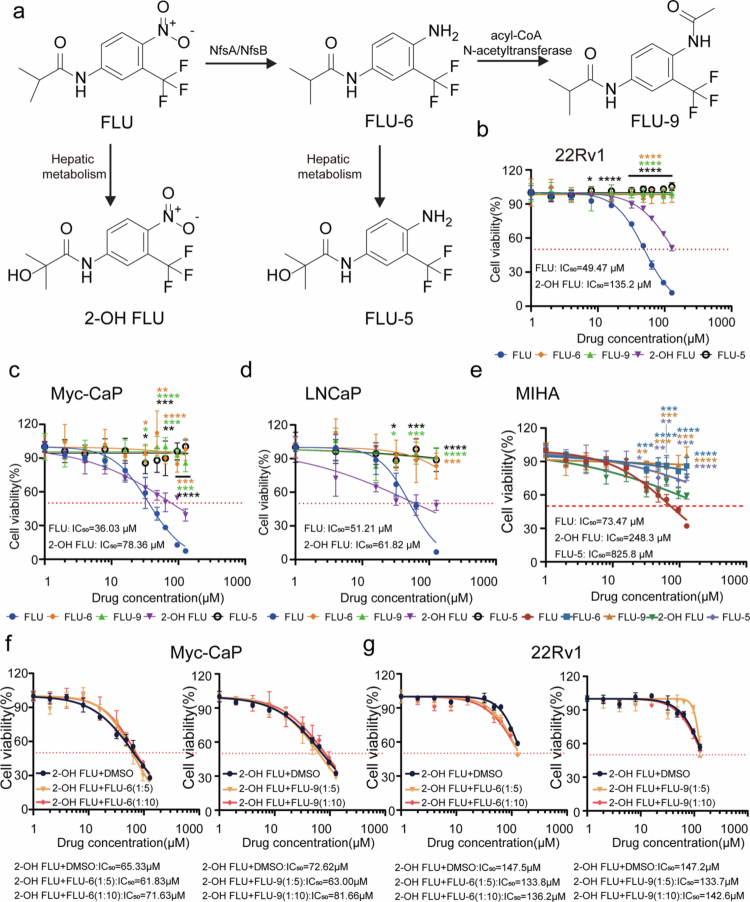
Lack of anti-prostate cancer activity in gut microbiota-derived Flutamide metabolites FLU-6, FLU-9, and FLU-5. (a) Chemical structures and proposed metabolic pathways of FLU and its metabolites. FLU is first metabolized by microbial nitroreductases NfsA and NfsB into FLU-6, which is subsequently converted into FLU-9 via microbial acyl-CoA *N*-acetyltransferase activity. Both FLU and FLU-6 can also undergo hepatic metabolism, yielding 2-OH FLU and FLU-5, respectively. (b-d) Effects of varying concentrations of FLU, FLU-6, FLU-9, 2-OH FLU, and FLU-5 on the proliferation of prostate cancer cell lines 22Rv1 (b), Myc-CaP (c), and LNCaP (d). Colored asterisks indicate comparisons between Flutamide and its metabolites: yellow for FLU-6, green for FLU-9, and black for FLU-5. (e) Cytotoxicity of Flutamide and its metabolites in human hepatocyte MIHA cells. Colored asterisks indicate comparisons between Flutamide and its metabolites: blue for FLU-6, yellow for FLU-9, and purple for FLU-5. (f, g) Cell viability of Myc-CaP (f) and 22Rv1 (g) prostate cancer cells treated with 2-OH FLU alone or in combination with FLU-6 or FLU-9 at ratios of 1:5 and 1:10, as measured by CCK-8 assay. IC_50_ values are indicated. Data are presented as mean ± SD, *n* = 5. *p < 0.05, **p < 0.01, ***p < 0.001, ****p < 0.0001.

We first summarized the above metabolites and their proposed metabolic pathways (Figure 4a), and further developed a strong interest in their pharmacological effects. Virtual molecular docking analysis of Flutamide and its metabolites with the Androgen receptor (AR) was conducted. It indicated attenuated binding affinity with the AR of microbiota-derived Flutamide metabolites, including FLU-6 (−5.29 kcal/mol), FLU-9 (−5.51 kcal/mol), as well as the liver-derived metabolite FLU-5 (−5.63 kcal/mol), compared with Flutamide (−7.82 kcal/mol) and 2-OH FLU (−8.14 kcal/mol), the results are shown in Supplementary Figure S5a-e.

We chemically synthesized FLU-6, FLU-9, and FLU-5, using Flutamide as the precursor^44^ (Supplementary Figure S5f-h), with structures confirmed by NMR spectroscopy (Supplementary Figure S6a-f), and purities >95% by HPLC (Supplementary Figure S6g-i). Antitumor activities evaluation in androgen-dependent prostate cancer cell lines (22Rv1, LNCaP, Myc-CaP) demonstrated: (1) all Flutamide metabolites (FLU-5/6/9) did not exhibit significant anti-proliferative effects; (2) both Flutamide and 2-OH FLU demonstrated potent antitumor activity (IC_50_ values: 36.03–51.21 μM of Flutamide, 61.82–135.2 μM of 2-OH FLU). These findings indicate gut bacterial metabolism diminished Flutamide's antitumor efficacy in prostate cancer cells (Figure 4b-d).

Given hepatotoxicity is a major adverse effect of Flutamide, we evaluated the cytotoxicity of Flutamide and its microbial metabolites in human hepatocyte MIHA cells. The IC_50_ value of FLU was 73.47 μM. In contrast, 2-OH FLU and FLU-5 displayed markedly reduced cytotoxicity, with IC_50_ values of 248.3 and 825.8 μM, respectively. Notably, both FLU-6 and FLU-9 exhibited negligible cytotoxicity within the tested concentrations. These findings suggest microbial transformation likely attenuates FLU's hepatotoxic potential (along with a decrease in pharmacological efficacy), particularly through the generation of FLU-6 and FLU-9 (Figure 4e). Meanwhile, we evaluated the impact of FLU-6 and FLU-9 on the pharmacological activity of the active metabolite 2-OH FLU in Myc-CaP and 22Rv1 cells. The results showed that these metabolites did not exhibit antagonistic or synergistic effects on the activity of 2-OH FLU (Figure 4f-g).

Furthermore, ADMETlab 3.0^45^ profiling of FLU-6 and FLU-9 indicated that these microbial metabolites may act as potential inhibitors of key drug transporters such as *P*-glycoprotein (Pgp),^46^ OATP1B3,^47^ and MRP1,^48^ suggesting a possible risk of drug-drug interactions despite their lack of cytotoxicity or antitumor activity (Supplementary Table S9).

### Compromised anti-prostate cancer effect of Flutamide after *E. coli* metabolism

**Figure 5.**
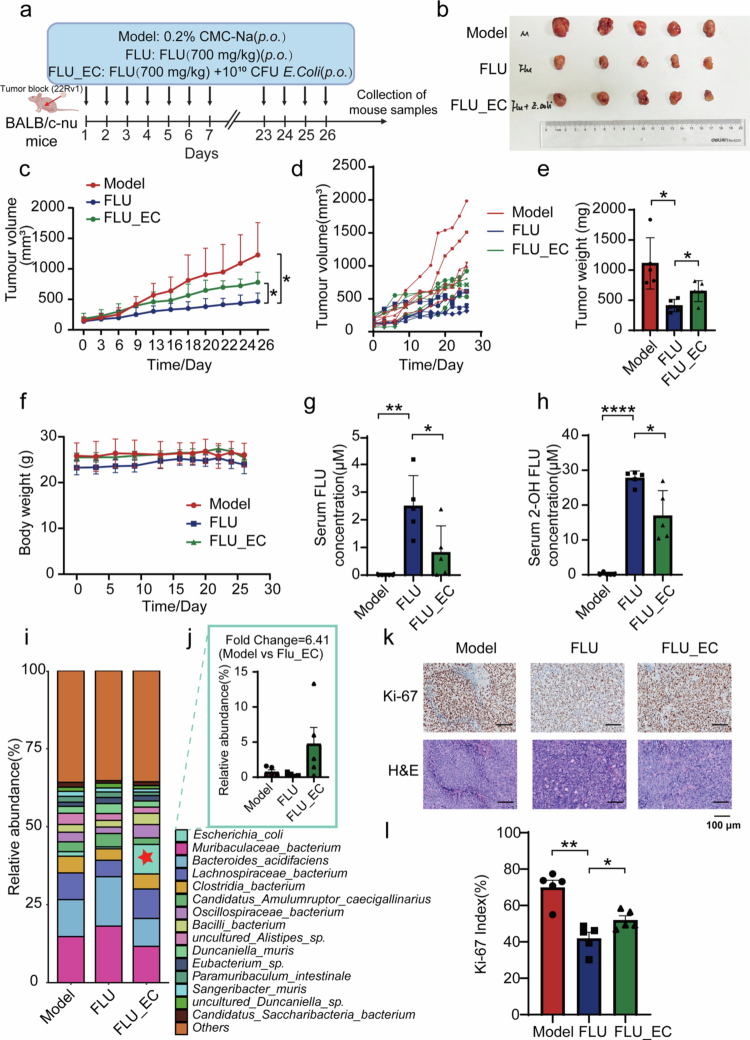
*E. coli*-mediated modulation of Flutamide antitumor efficacy. (a) Experimental grouping and schematic representation of the study design. (b) Photographs of tumors resected on day 26 post-treatment (*n* = 5 mice per group). (c) Tumor volume progression over time. (d) Individual tumor volume changes over time in the mice. (e) Tumor weight post-treatment across groups. (f) Body weight change in mice. (g, h) Serum concentrations of FLU (g) and 2-OH FLU (h) across groups. (i) Species abundance profiles in fecal samples from mice across groups, obtained from Shotgun metagenomic sequencing; each color represents a species. “Others” denotes the sum of all valid bacterial taxa at the species level falling outside the top 15 in relative abundance. (j) Abundance distribution of *E. coli* across the three groups based on Shotgun metagenomic sequencing. (k) Immunohistochemical analysis of 22Rv1 cell xenograft tumors (100× magnification). (l) Quantification of Ki-67 positive cells in tumor tissues from each group. Data are expressed as Ki-67 index (%). Data in panels (c-h, l) are presented as mean ± SD. Data in panel (j) are presented as mean ± SEM. Statistical significance was denoted as follows: *p < 0.05, **p < 0.01, ***p < 0.001, and ****p < 0.0001.

We evaluated the pharmacological activity of FLU alone or combined with *E. coli* in 22Rv1 prostate cancer-bearing BALB/c nude mice over 26 d. Tumor tissues, feces, and serum were collected for analysis (Figure 5a). The antitumor efficacy of Flutamide was diminished in the presence of *E. coli*, evidenced by increased tumor volume (1.68-fold) and weight (1.59-fold) in the FLU_EC group versus FLU alone (Figure 5b-e). *E. coli* administration showed no significant effect on body weight (Figure 5f). Serum concentrations of both Flutamide, and its active metabolite 2-OH FLU were reduced in the FLU_EC group compared to Flutamide monotherapy (Figure 5g-h). Shotgun Metagenomic sequencing of fecal samples revealed elevated *E. coli* abundance in the FLU_EC group versus controls, with 6.41-fold and 14.97-fold increases relative to the Model and FLU groups, respectively (Figure 5i-j). Correspondingly, nuclear Ki-67 expression, a proliferation marker in tumor cells, was significantly lower in the FLU group than in the other groups (Figure 5k-l).

### Interindividual variability of Flutamide metabolism caused by gut microbiota in patients with prostate cancer

**Figure 6.**
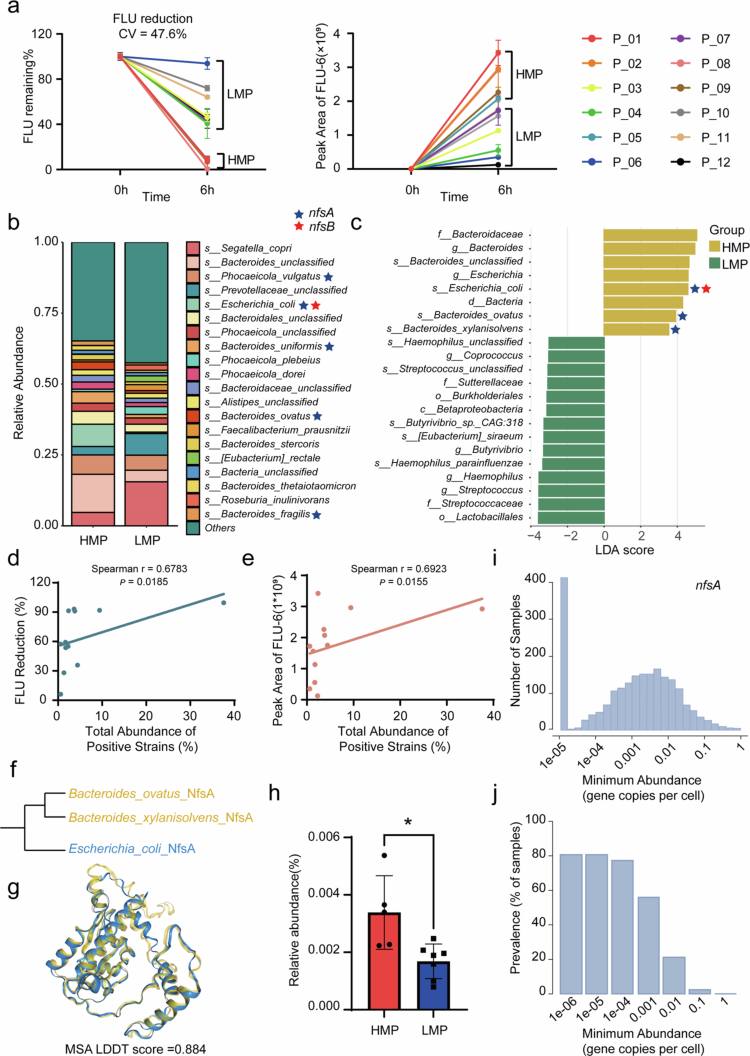
Interindividual variability in the metabolic capability of Flutamide. (a) FLU was incubated with fecal microbiota from different individuals. The levels of Flutamide and FLU-6 were measured at 0 and 6 h. (b) Comparison of the relative abundance of the top 20 gut bacterial species between HMP and LMP groups based on Shotgun metagenomic sequencing. Species whose representative genomes were predicted to encode proteins homologous to *E. coli* NfsA (UniProt ID: P17117) or NfsB (UniProt ID: P38489) are indicated by blue and red pentagrams, respectively. Homology was determined by BLASTP with the following criteria: query coverage >80%, E-value < 1e−10, and identity > 30%. “Others” denotes the sum of all valid bacterial taxa at the species level falling outside the top 20 in relative abundance. (c) LDA bar chart displaying species with significant intergroup differences (LDA score > 3) derived from Shotgun metagenomic data. Bar height indicates the magnitude of contribution to group separation. (d, e) Correlation analysis between the total abundance of NfsA/NfsB-harboring species (*E. coli*, *B*acteroides* ovatus*, and *B*acteroides* xylanisolvens*) and (d) Flutamide decrease or (e) FLU-6 increase. Spearman's correlation coefficients and *p*-values are shown. (f) Phylogenetic tree showing alignment of NfsA protein sequences from *E. coli*, *B. ovatus*, and *B. xylanisolvens*. (g) Predicted structural homology of NfsA. The structure of *E. coli* NfsA is represented in blue, whereas that of *B.ovatus* and *B.xylanisolvens* is shown in yellow. (h) Total abundance of *nfsA* and *nfsB* genes across different groups on gene-level annotations from Shotgun metagenomic sequencing. The x-axis indicates the sample groups (HMP and LMP), and the y-axis represents the combined relative abundance of *nfsA* and *nfsB* genes based on gene name annotations from Shotgun metagenomic sequencing data. (i) Abundance of *nfsA* homologs across human gut microbiome samples. (j) Prevalence of *nfsA* homologs across human gut metagenomes at different abundance thresholds. Data based on MetaQuery human metagenomic database. Data are presented as mean ± SD. *p < 0.05, **p < 0.01, ***p < 0.001, ****p < 0.0001.

Given substantial interindividual variation in gut microbiota composition, we assessed Flutamide metabolism profiles in gut microbiota from 12 prostate cancer patients (baseline characteristics: Supplementary Table S3 and S4). As illustrated in Figure 6a, notable heterogeneity in the Flutamide metabolic capacity was observed (Supplementary Table S10). Microbiota from five patients, which were defined as the high-metabolizer population (HMP), demonstrated high metabolite capacity (>90% Flutamide metabolism; median: 93.39%, range: 91.07%–99.58%) and elevated FLU-6 production (median peak area: 2.73 × 10^9^, range: 2.07 × 10^9^−3.42 × 10^9^). In contrast, microbiota from the remaining seven patients, defined as the low-metabolizer population (LMP), exhibited low metabolic capacity of Flutamide (median Flutamide metabolism: 42.18%, range: 6.14%–59.21%) and lower FLU-6 levels (median peak area: 1.03 × 10^9^, range: 1.24 × 10^8^–1.75 × 10^9^).

Metagenomic analysis was performed to explore gut microbes' involvement in Flutamide metabolism. Representative genomes of the top 20 most abundant bacterial species were searched for NfsA- and NfsB-like proteins using BLASTP. *E.coli* was found to carry both NfsA and NfsB homologs, whereas *Phocaeicola vulgatus*, *Bacteroides uniformis*, *Bacteroides ovatus,* and *Bacteroides fragilis* contained only NfsA homologs. Species harboring either NfsA or NfsB homologs were classified as nitroreductase-positive species. Their cumulative abundance among the top 20 most abundant species tended to be higher in the HMP versus LMP group (Figure 6b, Supplementary Table S11). Linear discriminant analysis (LDA) corroborated *E. coli, P. vulgatus*, and *B. xylanisolvens* as major discriminators (Figure 6c). A significant correlation was observed between the abundance of positive species and decreased Flutamide levels (Spearman's *r =* 0.678, *p =* 0.0185), and increased FLU-6 level (Spearman's *r =* 0.692, *p =* 0.0155) (Figure 6d-e). These findings indicate that the abundance of positive species may influence Flutamide metabolism, affecting FLU-6 production.

AlphaFold2^49^-predicted 3D protein structures and subsequent alignment revealed that substantial homology existed between *B.ovatus, B.xylanisolvens,* and *E. coli* NfsA protein (Multiple Sequence Alignment local Distance Difference Test score = 0.884, Figure 6f-g). To evaluate microbial nitroreductases functional capacity, we analyzed KEGG Orthology annotations from Shotgun metagenomic sequencing data. The relative abundances of *nfsA* and *nfsB* genes were extracted and summed. The abundance of these genes was significantly higher in the HMP group than in the LMP group (*p* < 0.05, Figure 6(h)), suggesting enhanced nitroreductase activity potential in the HMP group microbiome.

Compared to the *nfsB* gene, the *nfsA* gene exhibited a higher abundance within the human gut microbiota, with a distribution range primarily spanning from 0.001 (one copy per 1,000 cells) to 0.01 (one copy per 100 cells), based on data from the MetaQuery human metagenomic database (Figure 6i and Supplementary Figure S7a), and the *nfsA* gene was more prevalent in the human gut microbiota, with *nfsA* present in 77.6% of samples at a minimum abundance of 0.0001 (1 copy per 10,000 cells), compared to 69.6% for *nfsB* (Supplementary Figure S7b, Figure 6j)^50^. These data indicated that *nfsA* homologs and *nfsB* homologs are prevalent in human microbial populations.

## Discussion

Gut microbiota play a pivotal role in the pharmacokinetics of orally administered drugs by metabolizing them,^51^ leading to drug activation, reduced effect, and toxicity.^20^ In the present study, we demonstrate that gut microbiota impair the antitumor effect of Flutamide by directly metabolizing it via nitroreductases and acetyltransferases. We identified three gut microbiota-dependent metabolites lacking anticancer activity. Furthermore, we characterized the gut microbiota-mediated metabolic profile of Flutamide, revealing substantial interindividual variability. These findings indicate that gut microbiota may reduce Flutamide efficacy by metabolizing it into inactive compounds, and variations in microbial composition are likely to contribute to individual differences in drug metabolism.

Recent research has investigated the role of gut microbiota in androgen deprivation therapy (ADT) efficacy, revealing mechanisms involving the promotion of androgen biosynthesis and the modulation of immune function.^18^ ^,^ ^52^ ^,^ ^53^ While the involvement of gut microbiota in drug metabolism is well-established, the specific metabolic alterations and their consequences for ADT drugs remain unknown. A study investigated the *in vitro* metabolism profiles of abiraterone, an irreversible CYP17A1 inhibitor with antiandrogen activity in both hepatic and gut microbiota systems, and identified a bacteria-derived metabolite. The study provides a theoretical basis for the microbe-induced metabolic mechanism, and inspires new research ideas, particularly concerning the metabolism of other ADT drugs and the significant clinical implications of microbial metabolism. Our findings extend this concept by demonstrating that gut microbiota, particularly *E. coli*, directly metabolize Flutamide through nitroreductases, forming inactive products and reducing its therapeutic efficacy.

Prior to this study, microbial metabolism of Flutamide remained unexplored. All eight previously reported Flutamide metabolites, 2-OH FLU (active metabolite, via hepatic cytochrome P450 enzymes), FLU-1 and FLU-3 (via carboxylesterases), FLU-4, -5, -6, and -8 (via nitroreductase), were exclusively host enzyme-derived.^43^ Notably, host nitroreductases exhibit limited capacity to generate nitroreduction products under normoxic conditions, whereas under anaerobic conditions, the production rate of FLU-6 increases dramatically—by approximately 129-fold.^43^ Here, we identified a novel microbial-derived metabolite, *N*-(4-acetamido-3-(trifluoromethyl)phenyl) isobutyramide (designated as FLU-9), likely formed by acetyltransferase activity. Additionally, multiple gut bacterial strains produced FLU-6 (a nitroreduction product), which is further converted into FLU-5 through hepatic hydroxylation. These findings suggest that nitroreduction of Flutamide may originate from an alternative, abundant nonhepatic source, highlighting a typical host–microbiota cometabolic pathway. Crucially, these microbiota-derived metabolites lack antitumor activity, indicating that gut microbial metabolism significantly diminishes Flutamide's therapeutic efficacy.

Gut microbial metabolism of Flutamide involves both nitroreductases (NRs) and acetylation. Ubiquitous in gut microbiota, NRs critically metabolize nitro-containing drugs.^54^ For instance, gut bacterial NRs reduce nitrazepam to 7-aminonitrazepam, a metabolite with reduced anti-insomnia activity but increased teratogenic risk.^55^ Similarly, NRs reduce Flutamide's nitro group (-NO_2_) to an amino group, generating FLU-6. Acetylation, catalyzed primarily by gut bacterial acetyl-CoA *N*-acyltransferases (Bacteroidetes), typically inactivates drugs or increases toxicity (e.g., loss of therapeutic efficacy in acetylated 5-aminosalicylic acid). Heterologous expression confirmed that specific gut microbial acetyltransferases acetylate FLU-6, producing FLU-9. These findings provide novel insights into the mechanisms by which Flutamide bioavailability is reduced.

The core concern is how gut bacterial metabolism alters Flutamide's clinical response. The three microbial metabolites of Flutamide lack anti-proliferative effects on prostate cancer cells, and gut microbiota directly diminish Flutamide efficacy *in vivo*. Given the substantial interindividual variability in gut microbiota and its modulation by probiotics, antibiotics, or dietary interventions, it may potentially lead to treatment failure or significant efficacy variations. Patients were stratified by metabolic proportion: high-metabolizer patients (HMP, >90%) and low-metabolizer patients (LMP, <60%), showing a coefficient of variation (CV) exceeding 47.61%. *E. coli*, *B. ovatus*, and *B. xylanisolvens* were enriched in HMP, exhibited nitroreductase activity, and positively correlated with FLU-6 production. These findings propose that microbiome metabolism may serve as a potential basis for personalized Flutamide dosing. Probiotics, antibiotics, and dietary interventions can significantly modulate gut microbiota, thereby altering drug metabolism and efficacy. For example, coadministration of *Lactobacillus acidophilus* ^56^ ^,^ ^57^ reduces intestinal nitroreductase activity, or Vancomycin^58^ ^,^ ^59^ targeting Gram-positive bacteria. More targeted strategies—like transient antibiotic inhibition of specific drug-metabolizing bacteria (e.g., neomycin blocking *Eggerthella lenta*-mediated digoxin inactivation)^22^—offer promise. Targeted inhibition of microbiota-mediated metabolism is expected to enhance Flutamide efficacy.

While our findings highlight the impact of gut microbial metabolism (notably by *E. coli*) on Flutamide pharmacokinetics and treatment variability, the limited sample size may constrain generalizability. Validation in larger, diverse cohorts is needed to confirm these effects and support microbiota-based interventions, including the coadministration of microbial enzyme inhibitors,^57^ ^,^ ^60^ ^,^ ^61^ dietary modulation using structurally defined prebiotic fibers,^62^ and future application of gene-targeted microbial interventions.^63^ These approaches offer promising directions to enhance the efficacy of antiandrogen treatments through microbiota-informed precision strategies.^61^ ^,^ ^64^

## Conclusion

Flutamide metabolism is markedly modulated by the gut microbiota, particularly via metabolic pathways mediated by nitroreductases and acetyltransferases. Specific bacterial strains within the gut microbiota, such as *E. coli*, convert Flutamide into inactive metabolites, significantly diminishing its bioavailability and antitumor efficacy. Considerable interindividual variability in gut microbial composition among prostate cancer patients directly influences Flutamide metabolism. These findings provide novel mechanistic insights and suggest potential microbiome-targeted strategies for the precision and personalized management of Flutamide therapy.

## Supplementary Material

Supplementary_Materials clean.docx

Supplementary Tables.docx Supplementary Tables.docx

Author Affiliation Change Agreement.pdf Author Affiliation Change Agreement.pdf

## Data Availability

The raw reads of 16S rRNA gene sequencing and Shotgun metagenomic sequencing data were deposited in the GSA database (Accession Number: CRA020617, CRA031985, CRA032542). The raw data of metabolome deposited into the Iprox database (Accession Number: PXD058112). The code used in this study and all supporting data are available upon request.
