# Supplementary material for: Gut microbial metabolism of Flutamide attenuates its therapeutic efficacy against prostate cancer
Source: Gut Microbes. 2026 Jun 7;18(1):2682803. doi: 10.1080/19490976.2026.2682803 (PMC13248909; doi:10.1080/19490976.2026.2682803)
Supplement: Supplementary_Materials clean.docx [file KGMI_A_2682803_SM2941.docx]

Supporting Information

**Gut Microbial Metabolism of Flutamide Attenuates Its Therapeutic Efficacy Against Prostate Cancer**

Shujing Li^1,2†^, Haiying Ding^1,3†^, Jiaqi Wang^1†^, Lingjie Yuan^2^, Ying Zhou^1^, Weiben Xu^2^, Hang Yin^1^, Mengqian Ye^1^, Yuning Sha^1^, Fangyin Li^4^, Yousheng Liu^4^, Zhengqin Zhu^2^, Lulu Song^2^, Xiangyu Jin^1^, Liefeng Ma^2^, Zhajun Zhan ^2*^, Libin Pan ^1*^, Luo Fang ^1,5,6*^

^1^Department of Pharmacy, Zhejiang Cancer Hospital, Hangzhou Institute of Medicine (HIM), Chinese Academy of Sciences, Hangzhou, Zhejiang 310022, China

^2^College of Pharmaceutical Science, Zhejiang University of Technology, Hangzhou, Zhejiang 310022, China.

^3^Institute of Pharmacology and Toxicology, Zhejiang Province Key Laboratory of Anti-Cancer Drug Research, College of Pharmaceutical Sciences, Zhejiang University, Hangzhou 310022, China.

^4^Department of Urology, Zhejiang Cancer Hospital, Hangzhou Institute of Medicine (HIM), Chinese Academy of Sciences, Hangzhou, Zhejiang 310022, China.

^5^Hangzhou Institute of Medicine (HIM), Chinese Academy of Sciences, Hangzhou, Zhejiang 310022, China

^6^Postgraduate training base Alliance of Wenzhou Medical University (Zhejiang Cancer Hospital), Hangzhou, Zhejiang 310022, China

^†^Shujing Li, Haiying Ding and Jiaqi Wang contributed equally to this manuscript.

*Corresponding authors:

Luo Fang, [fangluo@zjcc.org.cn](mailto:fangluo@zjcc.org.cn);

Libin Pan, [panlb@zjcc.org.cn](mailto:panlb@zjcc.org.cn);

Zhajun Zhan, zjnpr@zjut.edu.cn

**Competing Interests:** The authors declare no potential conflicts of interest.

Materials and Methods

**Instrumentation and analytical methods for LC–MS/MS**

A high-performance liquid chromatography system coupled with a Q-Exactive Orbitrap mass spectrometer (Thermo Scientific, USA) was used to qualitatively analyze Flutamide and its metabolites in *ex vivo* co-incubation experiments with gut microbiota. Chromatographic separation was performed using an UltiMate 3000 UPLC system (Thermo Fisher Scientific, Bremen, Germany) equipped with an ACQUITY UPLC T3 column (100 mm × 2.1 mm, 1.8 μm; Waters, Milford, USA). The column temperature was maintained at 40 °C, and the flow rate was set at 0.3 mL/min. The mobile phases consisted of solvent A (5 mM ammonium acetate with 5 mM acetic acid in water) and solvent B (acetonitrile). The gradient elution profile was as follows: 0–0.8 min, 2% B; 0.8–2.8 min, 2–70% B; 2.8–5.6 min, 70–90% B; 5.6–6.4 min, 90–100% B; 6.4–8.0 min, 100% B; 8.0–8.1 min, 100–2% B; and 8.1–10 min, 2% B.

Mass spectrometric detection was performed using a Q-Exactive Orbitrap operated in both positive and negative ionization modes. Full MS spectra (*m/z* 70–1050) were acquired at a resolution of 70,000 with an AGC target of 3×10^6^ and a maximum injection time of 100 ms. Data-dependent acquisition (DDA) mode was used for MS/MS scans, collected at a resolution of 17,500 with an AGC target of 1×10^5^ and a maximum injection time of 80 ms. A pooled quality control sample was analyzed every 10 samples to assess system stability.

To evaluate the metabolic activity of various gut bacterial strains, including knockout and heterologous expression strains, as well as to quantify Flutamide and its metabolites in rat and mouse serum samples, a separate LC-MS/MS system was employed. This system comprised an HPLC system coupled to an Orbitrap Exploris^TM^ 120 mass spectrometer (Thermo Scientific). Chromatographic separation was performed on a C18 column (Kinetex®, Phenomenex, 2.1 × 100 mm, 2.6 μm; Part No. 00D-4462-AN) at 40 °C and a flow rate of 0.3 mL/min. The mobile phase consisted of phase A (6.5 mM ammonium acetate in water) and phase B (acetonitrile). The gradient was as follows: 0–0.5 min, 2% B; 0.5–2.0 min, 2–60% B; 2.0–4.5 min, 60–100% B; 4.5–5.0 min, 100% B; and 5.0–5.1 min, return to 2% B.

The Orbitrap Exploris^TM^ 120 was operated in negative ion mode. Full MS scans (*m/z* 60–900) were acquired at 60,000 resolution with an AGC target as per manufacturer’s protocol and a maximum injection time of 100 ms. MS/MS scans were acquired at 15,000 resolution with a maximum injection time of 100 ms.

Additional instruments included a refrigerated high-speed centrifuge (Thermo Fisher Scientific, USA), shaking incubator (Shanghai One Instrument Science Co. Ltd., China), and vortex mixer (Shanghai BaiDian Instrument Co. Ltd., China).

**NMR Spectroscopic Analysis**

^1^H (400 MHz) and ^19^F (376 MHz) NMR spectra were recorded on Bruker AVANCE NEO 400 and spectrometer with DMSO-d_6_ as solvent and tetramethylsilane (TMS) as the internal standard unless otherwise stated. All chemical shift values were reported in units of δ (ppm).

**Mass spectrometry data analysis for metabolite annotation**

Diverse bioinformatics tools can facilitate metabolite annotation. Accordingly, MS-DIAL and SIRIUS were used to identify putative fragments^[1]^. The LC-MS/MS data were obtained using the following methodology. The raw LC-MS/MS data were converted into Analysis Base File (ABF) format for peak alignment using MS-DIAL (Version 5.1). In MS-DIAL, the adduct ion dictionary is defined as follows: For data obtained from the negative ion mode, the following parameters were used: [M - H] and [M + Na - 2H]. The remaining parameter settings are as follows: retention time tolerance, 0.1 minutes; accurate mass tolerance (MS1), 0.01 Da; accurate mass tolerance (MS2), 0.025 Da; sigma window value, 0.5; and MS/MS abundance cut-off, 0 amplitude. This approach was used to determine metabolite abundance.

To facilitate the structural characterization of the detected features, MS/MS data were exported from the MS-DIAL in Mascot generic format (.mgf) file. This combined MGF file was then imported and analyzed using SIRIUS software (version 5.8.2)^[2, 3]^, which enabled the proposal of a molecular formula (SIRIUS) for a given feature and the elucidation of its structure based on fragmentation data (CSI:FingerID)^[4]^. The software was configured to analyze the mzML file with the following parameters: The instrument used was an Orbitrap, with the MS/MS isotope scorer set to "ignore" and the MS2 MassDev set to 10 ppm. Candidates were stored for 10 min, with at least one candidate per stored ion. The adducts were set as [M + Br]-, [M + Cl]-, and [M - H]-. The elemental composition was set as H, C, N, O, and F. The heuristic was used above *m/z* 300, and above *m/z* 650, it was used exclusively. All databases were searched, and the Canopus parameter was set to "parameter-free."

**Phylogenetic Analysis of Gut Bacterial Strains**

The 16S rRNA gene sequences of the 21 bacterial strains were obtained from the NCBI database. Multiple sequence alignment was performed using the MUSCLE algorithm in MEGA X with default parameters^[5]^. A phylogenetic tree was constructed using the maximum likelihood method, also in MEGA X, with 1,000 bootstrap replicates to assess tree robustness.

***In Vitro* Inhibition Assay of Flutamide Metabolism in *Escherichia coli***

For the overnight culture of *Escherichia coli* ATCC25922, following the measurement of the OD_600_, the culture was subjected to centrifugation at 5000 rpm for 10 min at 4 °C. The pellet was resuspended in PBS to an OD_600_ of 0.8. To each 594 μL culture, 3 μL of Flutamide stock solution and 3 μL of inhibitor stock solution at varying concentrations were added: menadione (final concentration 100 μM)^[6]^, 2-iodosobenzoic acid (final concentration 100 μM)^[7]^, and dicoumarol (final concentration 20 μM)^[8]^, resulting in a final Flutamide concentration of 50 μM. An equal volume of dimethyl sulfoxide (DMSO) was administered to the control group. The cultures were incubated at 37 °C with shaking at 200 rpm for 0 h and 6 h. Following the incubation period, an equal volume of ice-cold acetonitrile was added to terminate the reaction, and the samples were stored at -80 °C for subsequent analysis.

Following the incubation period, the samples were thawed on ice and 200 µL of each sample was mixed with an equal volume of ice-cold acetonitrile and vortexed for three minutes. Subsequently, the mixtures were allowed to stand at -20 °C for 1 h, after which they were subjected to centrifugation at 4 °C and 12,500 rpm for 10 min. A 10 μL aliquot of the supernatant was analyzed using HPLC coupled with an Orbitrap ExplorisTM 120 mass spectrometer to identify Flutamide metabolites.

**Phylogenetic analysis**

The protein sequences of NfsB and NfsA (UniProt IDs: P38489; P17117) were used in a BLASTp search against the reference and representative genome databases for bacteria and archaea, as provided by the Bacterial and Viral Bioinformatics Resource Center (BV-BRC). The alignment parameters were configured to allow for a maximum of 5,000 hits with an E-value threshold of 0.0001, facilitating the identification of significant matches. Sequences were selected based on the following criteria: Query Cover > 80%, E-value < 10^-10, and identity > 80%^[9]^. Duplicate and unclassified entries were excluded based on their annotation. To facilitate visualization, a maximum of five NfsB and NfsA homologs were selected from each genus. The selected protein sequences were aligned using the MUSCLE algorithm, implemented in MEGA Ⅹ^[5]^, with the default settings. A maximum likelihood phylogenetic tree was constructed in MEGA Ⅹ using the same default parameters, followed by 1,000 bootstrap replicates to assess the robustness of the tree. Taxonomic information within the phylogenetic tree was manually annotated using distinct colors for clarity.

**Serum** **and Bile Sample Preparation and LC-MS/MS Analysis**

Serum and Bile samples were thawed on ice, and 80 μL of each sample was mixed with three volumes of acetonitrile to precipitate proteins. The mixture was vortexed for 3 min and then incubated at -20 °C for 1 h. After incubation, samples were centrifuged at 12,500 rpm for 10 min at 4 °C. A 10 μL aliquot of the supernatant was analyzed by LC-MS/MS using the Orbitrap Exploris^TM^ 120.

**Fecal Sample Collection and Preparation**

Male C57BL/6 mice (SPF grade) were orally administered Flutamide at a dose of 90.1 mg/kg once daily for 3 consecutive days. On the third day, fecal samples were collected at multiple time points after dosing: 0-2 h, 2-4 h, 4-6 h, 6-8 h, 8-10 h, and 10-24 h. The feces were immediately snap-frozen on dry ice and lyophilized. A total of 0.1 g of freeze-dried fecal sample was homogenized with 4 mL of 50% methanol (w/v = 1:40). The homogenate was extracted at -20 °C for 1 h, followed by centrifugation at 12,500 rpm for 10 min at 4 °C. The supernatant was collected and centrifuged again under the same conditions to further clarify. A volume of 0.7 mL of the clarified supernatant was dried using a vacuum concentrator and reconstituted in 100 μL of 50% acetonitrile. The reconstituted samples were centrifuged at 12,500 rpm for 10 min at 4 °C, and a 10 μL aliquot of the supernatant was analyzed by LC-MS/MS using the Orbitrap Exploris^TM^ 120.

**Enterohepatic Circulation Assay**

To assess the potential enterohepatic recirculation of Flutamide and its metabolites, bile duct cannulation was performed in rats under isoflurane anesthesia. Following a midline abdominal incision, the duodenum was gently exteriorized, and the common bile duct was carefully isolated and ligated with surgical sutures. A polyethylene catheter was inserted into the bile duct and secured to ensure continuous bile drainage. The intestine was repositioned, and the abdominal cavity was covered with moist gauze to prevent dehydration. Bile was collected at 2-hour intervals over a total of 6 hours (0-2 h, 2-4 h, 4-6 h). Rats were continuously monitored and re-anesthetized as needed during the collection period. At the end of the experiment, animals were euthanized by cervical dislocation.

**Method description of 16S rRNA gene sequencing**

**DNA extractions**

DNA from different samples was extracted using CTAB according to manufacturer’s instructions. The reagent which was designed to extract DNA from trace amounts of sample has been shown to be effective for the preparation of DNA of most bacteria. Nuclear-free water was used for blank. The total DNA was eluted in 50 μL of Elution buffer and stored at -80 °C until measurement in the PCR by LC-Bio Technology Co., Ltd, Hang Zhou, Zhejiang Province, China.

**PCR amplification and 16S rRNA sequencing**

Genomic DNA was extracted from fecal samples, and the V3–V4 regions of the 16S rRNA gene were amplified using barcoded universal primers.The 5' ends of the primers were tagged with specific barcodes per sample and sequencing universal primers.PCR amplification was performed in a total volume of 25 μL reaction mixture containing 25 ng of template DNA, 12.5 μL PCR Premix, 2.5 μL of each primer, and PCR-grade water to adjust the volume. The PCR conditions to amplify the prokaryotic 16S fragments consisted of an initial denaturation at 98 ℃ for 30 seconds; 32 cycles of denaturation at 98 ℃ for 10 seconds, annealing at 54 ℃ for 30 seconds, and extension at 72 ℃ for 45 seconds; and then final extension at 72 ℃ for 10 minutes. The PCR products were confirmed with 2% agarose gel electrophoresis. Throughout the DNA extraction process, ultrapure water, instead of a sample solution, was used to exclude the possibility of false-positive PCR results as a negative control. The PCR products were purified by AMPure XT beads (Beckman Coulter Genomics, Danvers, MA, USA) and quantified by Qubit (Invitrogen, USA). The amplicon pools were prepared for sequencing and the size and quantity of the amplicon library were assessed on Agilent 2100 Bioanalyzer (Agilent, USA) and with the Library Quantification Kit for Illumina (Kapa Biosciences, Woburn, MA, USA), respectively. The libraries were sequenced on NovaSeq PE250 platform.

**Data analysis**

Samples were sequenced on an Illumina NovaSeq platform according to the manufacturer's recommendations, provided by LC-Bio. Paired-end reads were assigned to samples based on their unique barcode and truncated by cutting off the barcode and primer sequence. Paired-end reads were merged using FLASH. Quality filtering on the raw reads was performed under specific filtering conditions to obtain the high-quality clean tags according to the fqtrim (v0.94). Chimeric sequences were filtered using Vsearch software (v2.3.4). After dereplication using DADA2, we obtained feature table and feature sequences. Alpha diversity and beta diversity were calculated by normalized to the same sequences randomly. Then according to SILVA (release 138) classifier, feature abundance was normalized using relative abundance of each sample. Alpha diversity is applied in analyzing complexity of species diversity for a sample through 5 indices, including Chao1, observed species, Goods coverage, Shannon, Simpson, and all these indices in our samples were calculated with QIIME2. Beta diversity was calculated by QIIME2, the graphs were drawn by R package. Blast was used for sequence alignment, and the feature sequences were annotated with SILVA database for each representative sequence. Other diagrams were implemented using the R package (v3.5.2).

**General procedure for the preparation of FLU-6, FLU-9, FLU-5**

The chemical compounds Flutamide (2.4 mmol, 660 mg), B_2_(OH)_4_ (7.2 mmol, 648 mg), and 4,4'-bipyridine (0.012 mmol, 1.8 mg) were dissolved in 2 mL of dimethylformamide (DMF)^[10]^. The reaction mixture was then stirred at room temperature for 1 h. The reaction mixture was then diluted with water and ethyl acetate. The organic layer was collected and washed with brine. Subsequently, the solvent was evaporated under a reduced pressure. The crude product was purified by chromatography (petroleum ether-acetone = 8:1) to yield the compound designated as FLU-6.

**N-(4-amino-3-(trifluoromethyl) phenyl) isobutyramide (FLU-6)**

Yellow powder, yield: 49%;^1^H NMR (400 MHz, DMSO-d_6_): *δ* 9.67 (1H, s), 7.73 (1H, d, *J* = 2.5 Hz), 7.42 (1H, dd, *J* = 8.9, 2.4 Hz), 6.78 (1H, d, *J* = 8.8 Hz), 5.33 (2H, s), 2.52 (1H, m), 1.07 (6H, d, *J* = 6.8 Hz). ^13^C NMR (400 MHz, DMSO-d6): *δ* 175.1, 142.5 (d, *J* = 2.2 Hz), 128.8, 125.5 (q, *J* = 270.3 Hz), 125.5, 117.6, 117.4 (d, *J* = 5.5 Hz), 110.7 (q, *J* = 29.2 Hz), 35.2, 20.0 (C×2).

Acetyl chloride (142 μL, 2 mmol) was added dropwise to a solution of FLU-6 (246 mg, 1 mmol) and triethylamine (139 μL, 1 mmol) in dichloromethane (DCM) at room temperature for 2 hours. DCM was then evaporated, and the crude product was purified by chromatography (petroleum ether-acetone = 6:1) to yield compound FLU-9.

**N-(4-acetamido-3-(trifluoromethyl) phenyl) isobutyramide (FLU-9)**

White powder, yield: 62%;^1^H NMR (400 MHz, DMSO-d_6_): *δ* 10.15 (1H, s), 9.48 (1H, s), 8.10 (1H, d, *J* = 2.4 Hz), 7.78 (1H, dd, *J* = 8.9, 2.4 Hz), 7.37 (1H, d, *J* = 8.7Hz), 2.60 (1H, p, *J* = 6.8 Hz), 2.03 (3H, s), 1.12 (6H, d, *J* = 6.8 Hz). ^13^C NMR (400 MHz, DMSO-d_6_): *δ* 176.1, 169.7, 138.3, 131.6, 130.4, 125.7 (d, *J* = 23.3 Hz), 123.9 (q, *J* = 270.0 Hz), 123.3, 116.7, 35.5, 23.3, 19.8 (C×2).

The chemical compounds 3-hydroxyflutamide (1.2 mmol, 351 mg), B_2_(OH)_4_ (3.6 mmol, 324 mg), and 4,4'-bipyridine (0.006 mmol, 0.9 mg) were dissolved in dimethylformamide (DMF, 2 mL) in accordance with the experimental procedure. The reaction mixture was then stirred at room temperature for 1 h. The reaction mixture was then diluted with water and ethyl acetate. The organic layer was collected and washed with brine. Subsequently, the solvent was evaporated under a reduced pressure. The crude product was purified by chromatography (petroleum ether-acetone = 8:1) to yield compound FLU-5.

**N-(4-amino-3-(trifluoromethyl) phenyl)-2-hydroxy-2-methylpropanamide (FLU-5)**

Brown powder, yield: 37%;^1^H NMR (400 MHz, DMSO-d_6_): *δ* 9.45 (1H, s), 7.86 (1H, d, *J* = 2.5 Hz), 7.55 (1H, dd, *J* = 8.8, 2.4 Hz), 6.78 (1H, d, *J* = 8.8 Hz), 5.64 (1H, s), 5.35 (2H, s), 1.33 (6H, s). ^13^C NMR (400 MHz, DMSO-d6): *δ* 175.5, 142.8 (d, *J* = 2.4 Hz), 128.1, 126.1, 125.5 (q, *J* = 270.2 Hz), 118.0 (d, *J* = 5.8 Hz), 117.4, 110.4 (q, *J* = 20.9 Hz), 72.7, 28.2 (C×2).

**Flutamide molecular virtual docking analysis**

A docking analysis of Flutamide and its metabolites (FLU-6, FLU-9, 2-OH FLU, and FLU-5) with the AR was conducted using AutoDockTool software. The atomic structure of the AR was obtained from the Protein Data Bank (PDB) database. The three-dimensional docking models of Flutamide and its four metabolites with the AR were visualized using the PyMOL software. The parameters were set to their respective default values.

**ADMET Prediction Analysis**

The ADMET properties of Flutamide metabolites FLU-6 and FLU-9 were predicted using the online platform DMETlab 3.0 (https://admetlab3.scbdd.com/). This tool predicts absorption, distribution, metabolism, excretion, and toxicity characteristics based on chemical structure. Particular attention was given to potential interactions with drug transporters, including P-glycoprotein (P-gp), a key efflux transporter involved in multidrug resistance.

**Sample collection and measurement**

Fecal, serum, and tissue samples were collected from nude mice under sterile conditions to minimize contamination. On day 26, each mouse was transferred to a clean cage to collect fresh fecal samples. Following the collection of blood via the retro-orbital sinus, the samples were left to stand at room temperature for 1 h and then centrifuged at 5,000 rpm for 15 min. The supernatant was then meticulously separated to obtain serum samples. Following the collection of blood samples, mice were euthanized via cervical dislocation. Subsequently, the mice were dissected, and the tumor tissues were meticulously extracted. Tumor tissues were separated into two portions. One portion was fixed in paraformaldehyde for subsequent hematoxylin and eosin (HE) staining and Ki-67 detection, whereas the other was rapidly frozen on dry ice for temporary storage. The fecal samples were subjected to Shotgun metagenomic sequencing, and the serum, and half of the tumor tissues were stored at -80 °C until further analysis.

**Immunohistochemistry**

The excised tumors were subjected to a fixation process using 4% neutral buffered paraformaldehyde, after which they underwent a paraffin embedding procedure and were sectioned for subsequent immunohistochemical analysis. Cell proliferation was assessed using Ki-67 antibody (Proteintech, 27309-1-AP). Tissue morphology was observed using HE staining.

**Shotgun Metagenomic Sequencing and Analysis of Fecal Samples from Prostate Cancer Patients**

The total DNA was extracted from samples using the Fecal Genome DNA Extraction Kit (AU46111-96, BioTeke, China). DNA libraries were constructed using the TruSeq Nano DNA Library Preparation Kit-Set (#FC-121–4001, Illumina, USA) following the manufacturer’s instructions. Metagenome libraries were sequenced on an Illumina NovaSeq 6000 platform with PE150 at LC-Bio Technology Co., Ltd. (Hangzhou, China).

Fastp software (v0.23.4) was used to trim adaptor sequences, remove low-quality and ambiguous reads, and assess sequence quality. Quality-filtered reads were aligned to the human reference genome (hg38) using Bowtie2 (v2.2) to filter out host contaminations. The remaining reads were subjected to de novo assembly for each sample using MEGAHIT (v1.2.9).

MetaGeneMark (v3.26) was used to predict coding regions (CDS) from the assembled contigs, and CDS sequences of all samples were clustered using MMseq2 (v15-6f452) to obtain unigenes. DIAMOND (v0.9.14) was used to perform taxonomic assessment based on the NR database. The Wilcoxon test was used to identify differentially abundant species, with significance defined as P < 0.05 and |log2-fold change| > 1. Microbial function annotation was performed using the Kyoto Encyclopedia of Genes and Genomes (KEGG) database.


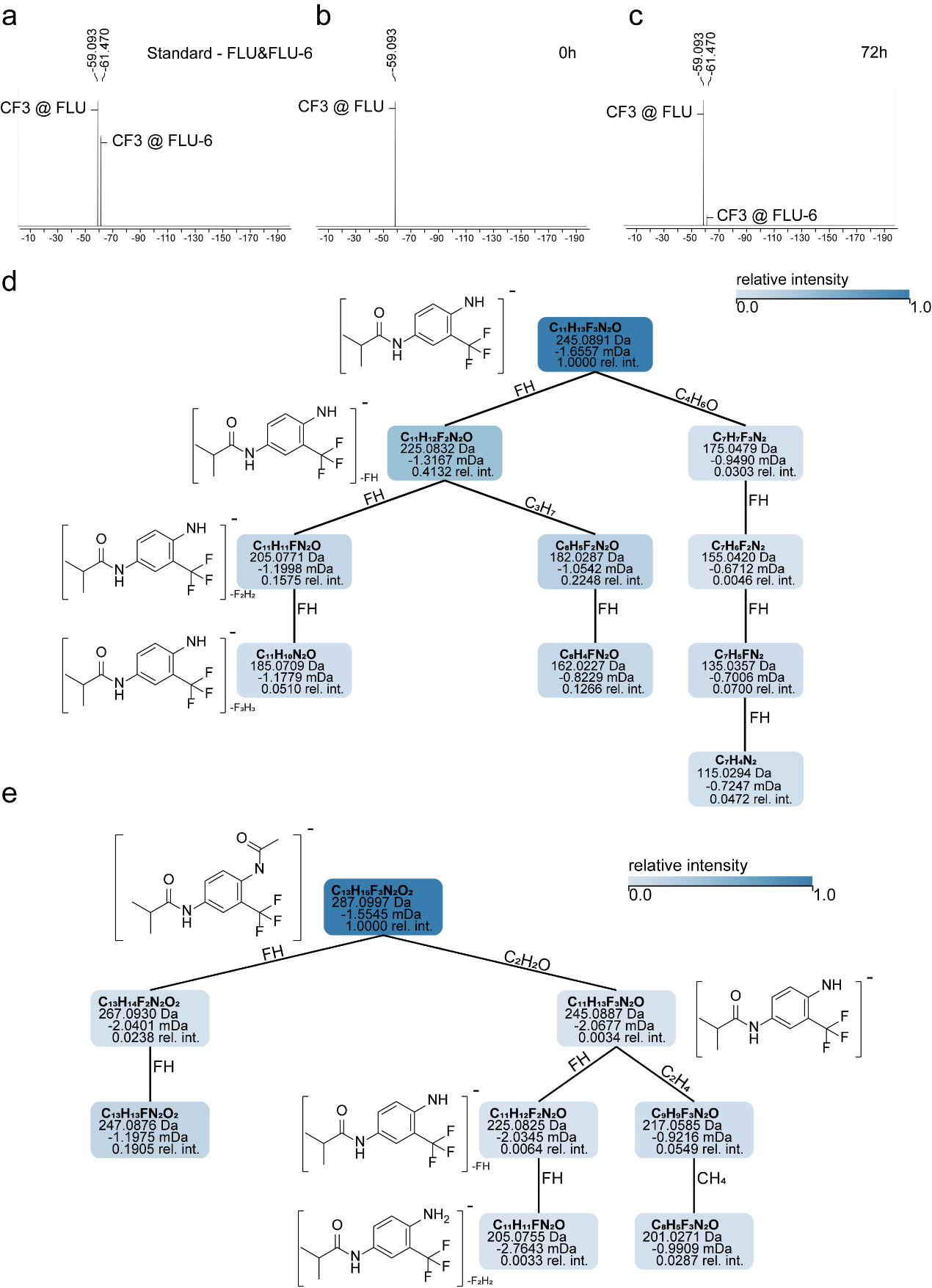


**Supplementary Figure S1. Structural Characterization and Fragment Ion Analysis of Flutamide Metabolites FLU-6 and FLU-9.** (a) F-spectra of standard compounds of Flutamide (FLU) and its metabolite FLU-6. (b, c) F-spectra of FLU incubated in an *ex vivo* mixed culturing system with gut microbiota at 0 h(b) and 72 h(c). (d, e) Fragmentation trees and proposed mass spectrometric cleavage pathways of metabolites FLU-6(d) and FLU-9(e) generated from LC-MS data, analyzed using SIRIUS software. Shared fragment ions at *m/z* 245.09, 225.08, 205.08, and 185.07 indicate common structural features between FLU-6 and FLU-9.

**
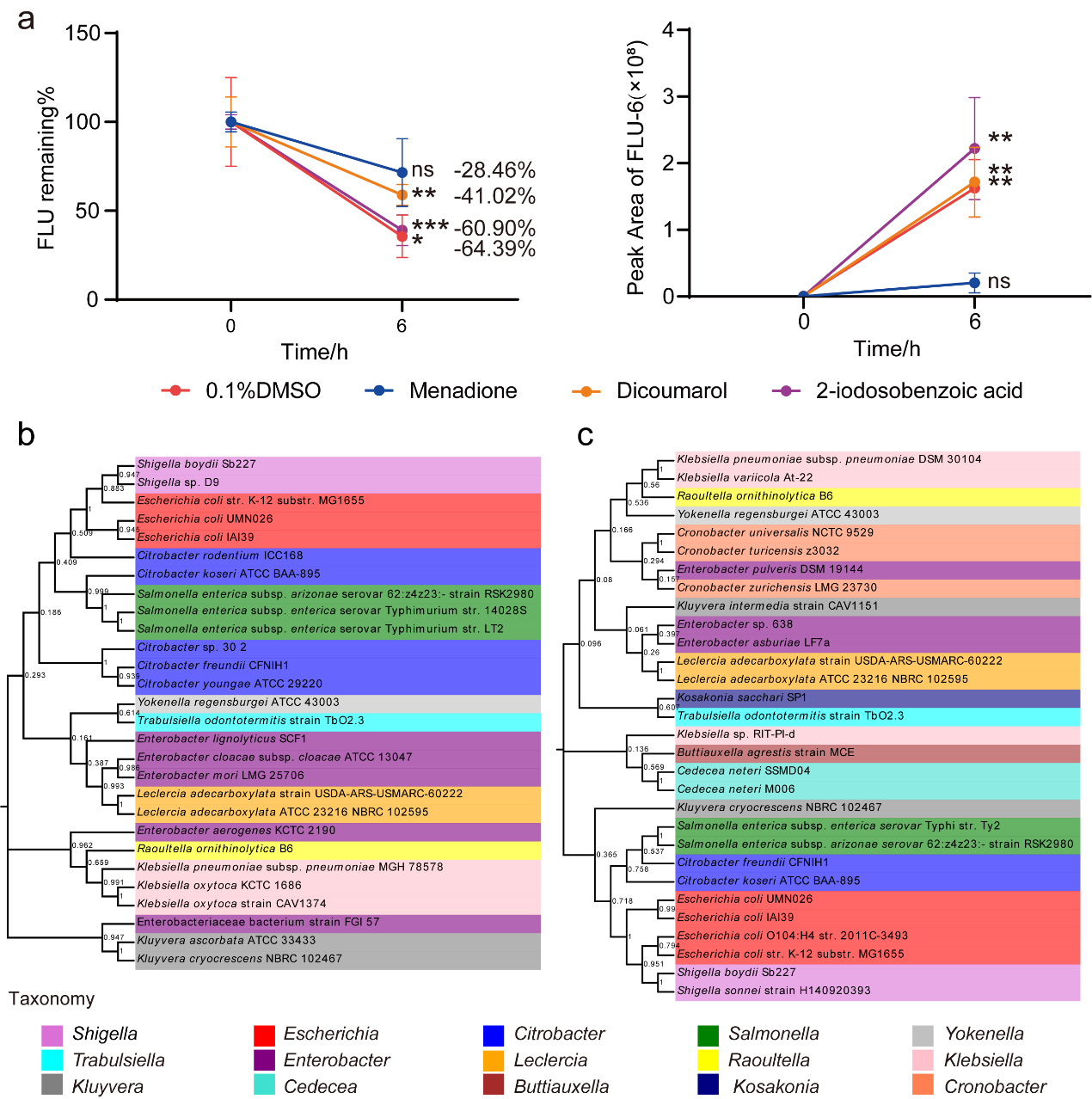
**

**Supplementary Figure S2. Inhibition of FLU metabolism by nitroreductase inhibitors and phylogenetic analysis of NfsA/NfsB homologs.** (A) Metabolic curves of FLU and FLU-6 in *Escherichia coli* treated with various nitroreductase inhibitors. (B) Maximum likelihood phylogenetic tree of NfsB homologs from different species and genera. (C) Maximum likelihood phylogenetic tree of NfsA homologs from different species and genera. The branch numbers represent bootstrap support values. All data are presented as the means ± SD. ****p* < 0.001, ***p* < 0.01, **p* < 0.05, and ns (not significant), all comparisons versus 0 h. Flutamide levels in WT *Escherichia coli* were reduced by 28.46%, 41.02%, 60.90%, and 64.39% under the treatment of Menadione, Dicoumarol, 2-iodosobenzoic acid, and solvent control (0.1% DMSO), respectively.


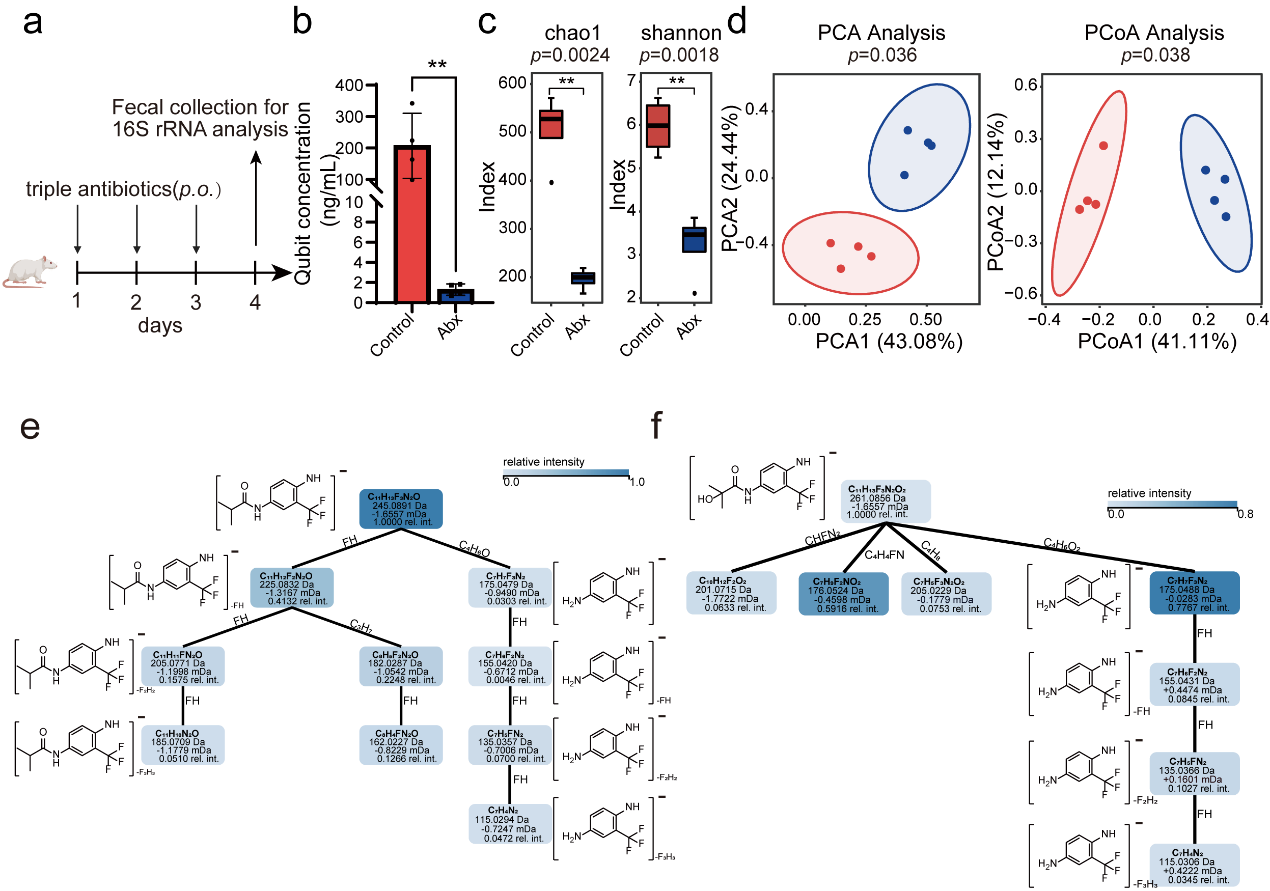


**Supplementary Figure S3.** **Antibiotic-induced microbiota removal and Fragment Ion Analysis of Flutamide Metabolites FLU-6 and FLU-5.** (a) Schematic diagram illustrating oral (*p.o.*) administration of a triple antibiotic in rats for 3 consecutive days to deplete gut microbiota prior to fecal sample collection for 16S rRNA gene sequencing. (b) Qubit concentration (ng/mL) in the control and Abx groups. (c) Effect of antibiotics on the α-diversity of gut bacteria in SD rats: Chao1 and Shannon indices based on 16S rRNA gene sequencing data. (d) Analysis of β-diversity after antibiotic treatment: Principal Component Analysis (PCA) and Principal Coordinates Analysis (PCoA), calculated from 16S rRNA gene sequencing results. (e, f) Fragmentation trees and proposed mass spectrometric cleavage pathways of metabolites FLU-6 (e) and FLU-5 (f) generated from LC-MS data of SD rats serum, analyzed using SIRIUS software. Shared fragment ions at *m/z* 175.05, 155.04, 135.04, and 115.03 are common to both FLU-6 and FLU-5.


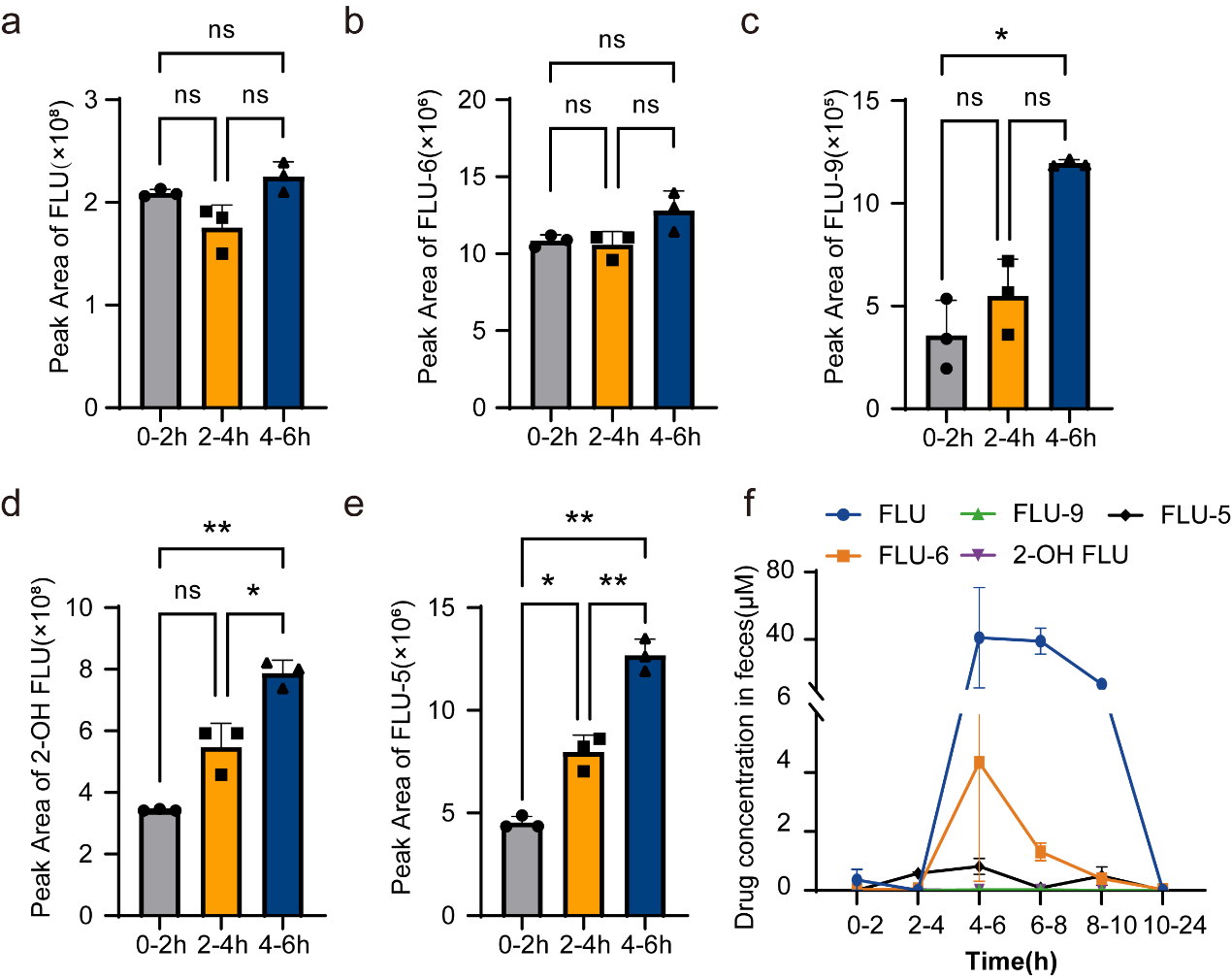


**Supplementary Figure S4. Biliary and fecal analysis of Flutamide and its major metabolites.**

(a–e) Peak areas of Flutamide (FLU, a) and its major metabolites—including FLU-6(b), FLU-9(c), 2-OH FLU(d), and FLU-5(e)—in bile samples collected from bile duct–cannulated rats following oral administration of flutamide. (f) Concentration (μM) of flutamide and its metabolites (FLU-6, FLU-9, FLU-5, and 2-OH FLU) in feces from C57BL/6 mice treated with oral flutamide (90.1 mg/kg/day) for three consecutive days. Data in panels (a-e) are presented as mean ± SD (n = 3). Data in panels (f) are presented as mean ± SEM (n = 3). **p* < 0.05; ***p* < 0.01; ns, not significant.


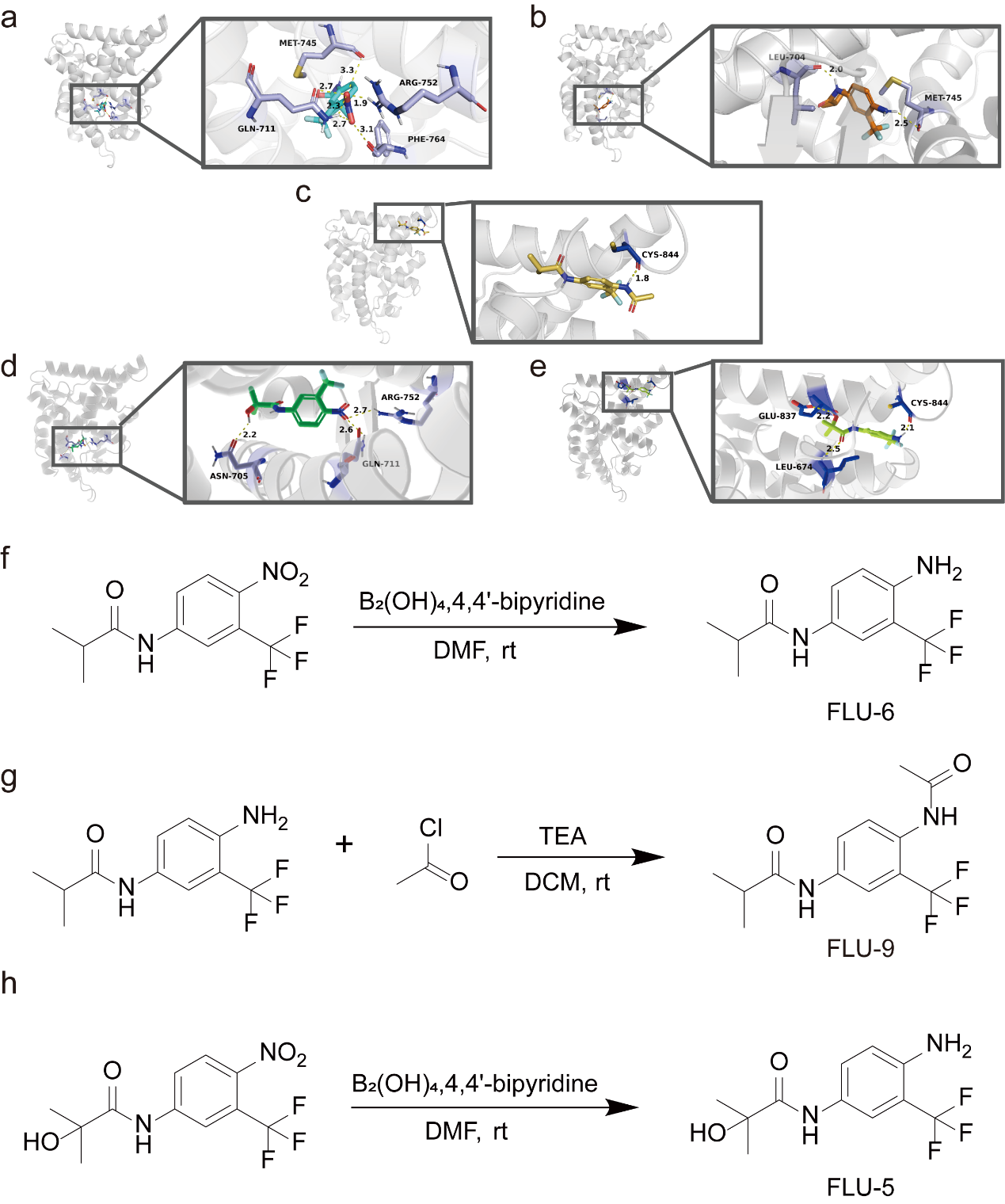


**Supplementary Figure S5. Molecular docking analysis and chemical synthesis routes.** (a-e) Molecular docking analysis of the androgen receptor with Flutamide (a), FLU-6 (b), FLU-9 (c), 2-OH FLU (d), and FLU-5 (e). (f-h) Synthesis pathways of metabolites FLU-6 (f), FLU-9 (g), and FLU-5 (h).


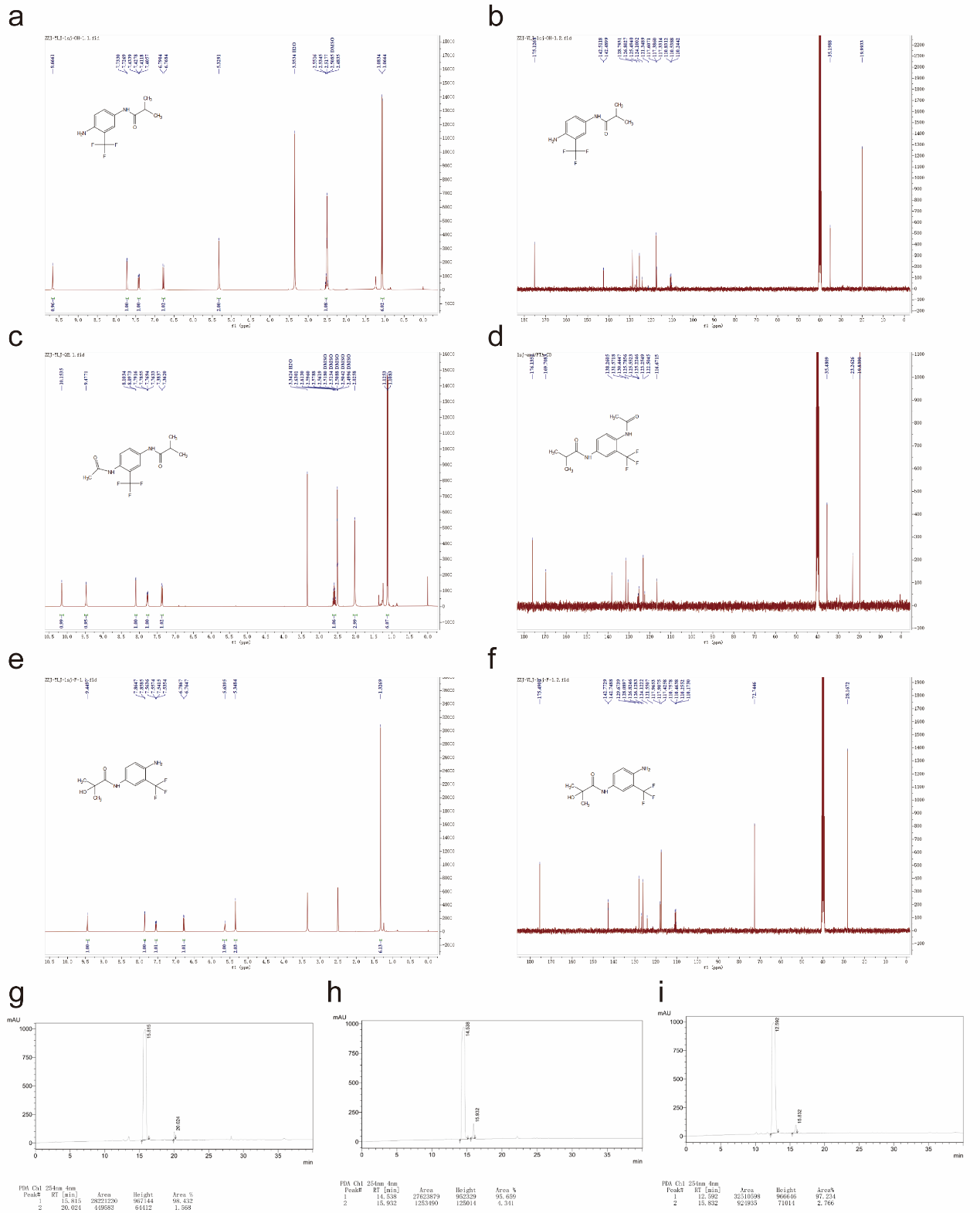


**Supplementary Figure S6.** **Spectral Characterization and Purity Assessment of Metabolites FLU-6, FLU-9, and FLU-5.** (a) ^1^H-NMR spectrum of FLU-6. (b) ^13^C-NMR spectrum of FLU-6. (c) ^1^H-NMR spectrum of FLU-9. (d) ^13^C-NMR spectrum of FLU-9. (e) ^1^H-NMR spectrum of FLU-5. (f) ^13^C-NMR spectrum of FLU-5. (g) Purity of FLU-6 determined by HPLC analysis. (h) Purity of FLU-9 determined by HPLC analysis. (i) Purity of FLU-5 determined by HPLC analysis.


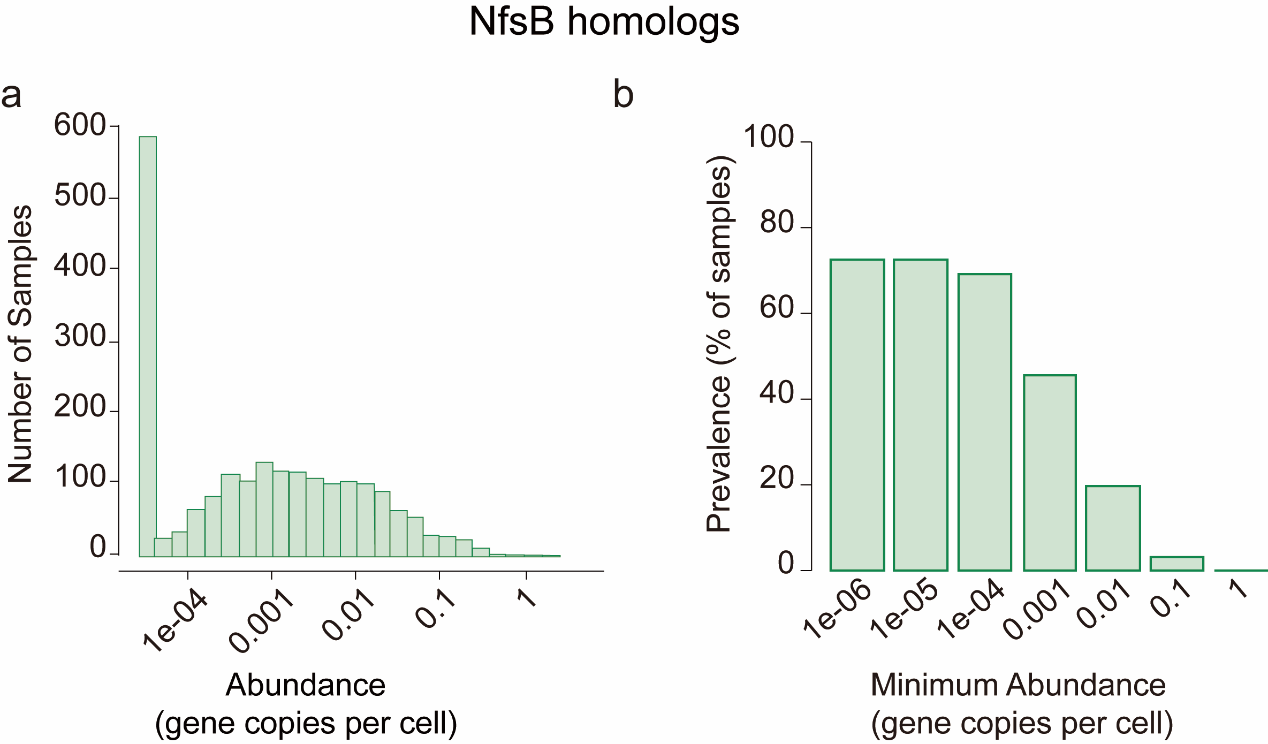


**Supplementary Figure S7. Abundance and prevalence of NfsB homologs in the human gut microbiome.** (a) Abundance of NfsB homologs across human gut microbiome samples. (b) Prevalence of NfsB homologs across human gut metagenomes at different abundance thresholds. Data based on MetaQuery human metagenomic database.

[1] SMITH E, LEWIS A, NARINE S S, et al. Unlocking potentially therapeutic phytochemicals in capadulla (doliocarpus dentatus) from guyana using untargeted mass spectrometry-based metabolomics [J]. Metabolites, 2023, 13(10).

[2] DüHRKOP K, FLEISCHAUER M, LUDWIG M, et al. Sirius 4: A rapid tool for turning tandem mass spectra into metabolite structure information [J]. Nat Methods, 2019, 16(4): 299-302.

[3] BöCKER S, DüHRKOP K. Fragmentation trees reloaded [J]. J Cheminform, 2016, 8: 5.

[4] DüHRKOP K, SHEN H, MEUSEL M, et al. Searching molecular structure databases with tandem mass spectra using csi:Fingerid [J]. Proc Natl Acad Sci U S A, 2015, 112(41): 12580-5.

[5] KUMAR S, STECHER G, LI M, et al. Mega x: Molecular evolutionary genetics analysis across computing platforms [J]. Mol Biol Evol, 2018, 35(6): 1547-9.

[6] RAFIL F, FRANKLIN W, HEFLICH R H, et al. Reduction of nitroaromatic compounds by anaerobic bacteria isolated from the human gastrointestinal tract [J]. Appl Environ Microbiol, 1991, 57(4): 962-8.

[7] LINWU S W, SYU C J, CHEN Y L, et al. Characterization of escherichia coli nitroreductase nfsb in the metabolism of nitrobenzodiazepines [J]. Biochem Pharmacol, 2009, 78(1): 96-103.

[8] ROLDáN M D, PéREZ-REINADO E, CASTILLO F, et al. Reduction of polynitroaromatic compounds: The bacterial nitroreductases [J]. FEMS Microbiol Rev, 2008, 32(3): 474-500.

[9] TIAN J, LI C, DONG Z, et al. Inactivation of the antidiabetic drug acarbose by human intestinal microbial-mediated degradation [J]. Nat Metab, 2023, 5(5): 896-909.

[10] JANG M, LIM T, PARK B Y, et al. Metal-free, rapid, and highly chemoselective reduction of aromatic nitro compounds at room temperature [J]. J Org Chem, 2022, 87(2): 910-9.
