# Supplementary material for: Gut microbial metabolism of Flutamide attenuates its therapeutic efficacy against prostate cancer
Source: Gut Microbes. 2026 Jun 7;18(1):2682803. doi: 10.1080/19490976.2026.2682803 (PMC13248909; doi:10.1080/19490976.2026.2682803)
Supplement: Supplementary Tables.docx [file KGMI_A_2682803_SM2939.docx]

| **Table S1: Culture Conditions and Strain Sources of Common Gut Microbial Species** | | | | | |
| --- | --- | --- | --- | --- | --- |
| Species | Strain | Genus | Culture Medium | Incubation Conditions | Strain sources |
| *Bifidobacterium longum* subsp. *infantis* | *Bifidobacterium longum* subsp. *infantis*  CGMCC 1.15639 | *Bifidobacterium* | mPYG | Anaerobic,37°C | NTB^1^ |
| *Bifidobacterium dentium* | *Bifidobacterium dentium*  ATCC 27534 |  | mPYG | Anaerobic,37°C | NTB^1^ |
| *Bacteroides thetaiotaomicron* | *Bacteroides thetaiotaomicron*  GDMCC1.1104 | *Bacteroides* | mPYG | Anaerobic,37°C | NTB^1^ |
| *Bacteroides fragilis* | *Bacteroides fragilis* NCTC 9343 |  | mPYG | Anaerobic,37°C | Clinical stool^2^ |
| *Bacteroides caccae* | *Bacteroides caccae* strain JCM 9498 |  | mPYG | Anaerobic,37°C | Clinical stool^2^ |
| *Bacteroides ovatus* | *Bacteroides ovatus* strain JCM 5824 |  | mPYG | Anaerobic,37°C | Clinical stool^2^ |
| *Bacteroides vulgatus* | *Bacteroides vulgatus* ATCC 8482 |  | mPYG | Anaerobic,37°C | NTB^1^ |
| *Bacillus altitudinis* | *Bacillus altitudinis* 41KF2b | *Bacillus* | mPYG | Anaerobic,37°C | Clinical stool^2^ |
| *Blautia obeum* | *Blautia obeum* DSM 25238 | *Blautia* | mPYG | Anaerobic,37°C | NTB^1^ |
| *Collinsella aerofaciens* | *Collinsella aerofaciens* strain JCM 10188 | *Collinsella* | mPYG | Anaerobic,37°C | Clinical stool^2^ |
| *Escherichia coli* | *Escherichia coli* ATCC 25922 | *Escherichia* | LB | Facultative anaerobic, 37°C | NTB^1^ |
| *Enterobacter quasihormaechei* | *Enterobacter quasihormaechei*  strain WCHEs120003 | *Enterobacter* | mPYG | Anaerobic,37°C | Clinical stool^2^ |
| *Enterococcus faecalis* | *Enterococcus faecalis* ATCC 51299 | *Enterococcus* | mPYG | Anaerobic,37°C | NTB^1^ |
| *Fusobacterium hwasookii* | *Fusobacterium hwasookii* ChDC F128 | *Fusobacterium* | mPYG | Anaerobic,37°C | NTB^1^ |
| *Fusobacterium nucleatum* | *Fusobacterium nucleatum* ATCC 25586 |  | mPYG | Anaerobic,37°C | NTB^1^ |
| *Lactobacillus acidophilus* | *Lactobacillus acidophilus* ATCC 4356 | *Lactobacillus* | mPYG | Anaerobic,37°C | NTB^1^ |

(**Table S1_continued)**

| *Peptostreptococcus anaerobius* | *Peptostreptococcus anaerobius* ATCC 27337 | *Peptostreptococcus* | mPYG | Anaerobic,37°C | NTB^1^ |
| --- | --- | --- | --- | --- | --- |
| *Parabacteroides merdae* | *Parabacteroides merdae* ATCC 43184 | *Parabacteroides* | mPYG | Anaerobic,37°C | NTB^1^ |
| *Ruminococcus gnavus* | *Ruminococcus gnavus* ATCC 29149 | *Ruminococcus* | mPYG | Anaerobic,37°C | NTB^1^ |
| *Streptococcus salivarius* | *Streptococcus salivarius* strain ATCC 7073 | *Streptococcus* | mPYG | Anaerobic,37°C | NTB^1^ |
| *Klebsiella pneumoniae* | *Klebsiella pneumoniae* ATCC 13882 | *Klebsiella* | mPYG | Anaerobic,37°C | NTB^1^ |

Abbreviations: mPYG, mPYG medium; LB, Luria-Bertani medium; NTB, Ningbo Taisituo Biotechnology Co., Ltd.

^1^ Strains obtained from NTB were purchased as reference strains from Ningbo Taisituo Biotechnology Co., Ltd.

^2^ Strains labeled as Clinical stool were isolated from human fecal samples.

| **Table S2: Primers Used for CRISPR/Cas9-Mediated Knockout of *nfsA* and *nfsB* in *E. coli*** | |
| --- | --- |
| Primer Name | Primer Sequence (5'-3') |
| pTB1-F | GTCCTAGGTATAATACTAGTAAACAGGTTTATCTCAACGTGTTTTAGAGCTAGAAATAGC |
| pTB1-R | TCAAAAAAAGCACCGACTCG |
| pTB2-F | CGAGTCGGTGCTTTTTTTGACATACTTTGCTTATTCTCCTTCGC |
| pTB2-R | GCAAGGGAGAAAAAGACTCCATGTGAAAGTAATTTTGC |
| pTB3-F | GGAGTCTTTTTCTCCCTTGCCGGGCAT |
| pTB3-R | TCTAAGCTTCTGCAGGTCGACGCCGCCGGGAATTTCTGC |
| pTA1-F | GTCCTAGGTATAATACTAGTAATAATCCAGAATAAATGGGGTTTTAGAGCTAGAAATAGC |
| pTA1-R | TCAAAAAAAGCACCGACTCG |
| pTA2-F | CGAGTCGGTGCTTTTTTTGACAATTTCTCGGCCAGATCTTTG |
| pTA2-R | CATCGACGTGGCAGTTTATCTTTTTCTCTTTCTGAACGTGAAT |
| pTA3-F | GATAACTGCCACGTCGATGTATGATAC |
| pTA3-R | TCTAAGCTTCTGCAGGTCGAC TGCGTGCCTTCAACCAGC |
| *nfsB*-F | CGACTGCGTCCTGACTCAAC |
| *nfsB*-R | ACCAGAATCGCGCCGATCAG |
| *nfsA*-F | CCCCACAGCTGATGAACCATCC |
| *nfsA*-R | TCGCAGCGACTACTTCATCGCC |

Abbreviations: F, Forward primer; R, Reverse primer.

Primers prefixed with 'pTA' and 'pTB' were used for cloning the single-guide RNA (sgRNA) expression cassettes targeting the *nfsA* and *nfsB* genes, respectively. Primers prefixed with 'nfsA-' and 'nfsB-' were used for PCR verification of the final knockout strains.

| **Table S3: Basic Characteristics of Prostate Cancer Patients** | | | | | | | | | | | | | | |
| --- | --- | --- | --- | --- | --- | --- | --- | --- | --- | --- | --- | --- | --- | --- |
| No. | Sex | Age  (years) | BMI (kg/m^2^) | Medical History | Gleason  Score | Gleason  Grade Group | PSA Level  (ng/mL) | Family history | Smoking | Drinking | Weight loss | T | N | M |
| P_01 | Male | 71 | 23.66 | Hypertension | 4 + 4 = 8 | 4 | 54.00 | Yes | Yes | Yes | No | pT4 | pN1 | pM1 |
| P_02 | Male | 64 | 30.49 | None | 3 + 4 = 7 | 2 | NA | No | Yes | Yes | No | pT2 | pNx | M0 |
| P_03 | Male | 55 | 25.10 | Hypertension | 3 + 4 = 7 | 2 | 11.69 | No | No | No | No | pT3a | pNx | M0 |
| P_04 | Male | 55 | 28.13 | None | 4 + 3 = 7 | 3 | 4.24 | Yes | No | No | No | pT2 | pNx | M0 |
| P_05 | Male | 68 | 28.51 | None | 3 + 4 = 7 | 2 | 10.00 | No | No | No | No | pT2 | pNx | M0 |
| P_06 | Male | 70 | 29.69 | None | 4 + 3 = 7 | 3 | 9.61 | No | Yes | Yes | No | pT3a | pN0 | cM1 |
| P_07 | Male | 74 | 23.32 | None | 4 + 4 = 8 | 4 | 8.70 | No | No | No | No | pT2 | pNx | M0 |
| P_08 | Male | 77 | 18.29 | None | 4 + 3 = 7 | 3 | 33.8 | No | No | Yes | No | pT3a | pNx | M0 |
| P_09 | Male | 62 | 22.86 | None | 3 + 3 = 6 | 1 | 6.00 | No | Yes | Yes | No | pT2 | pNx | M0 |
| P_10 | Male | 76 | 26.73 | None | 3 + 4 = 7 | 2 | 3.16 | No | Yes | Yes | No | pT2 | pNx | M0 |
| P_11 | Male | 69 | 18.36 | None | 3 + 5 = 8 | 4 | 43.30 | No | No | No | No | pT3a | pN0 | M0 |
| P_12 | Male | 75 | 25.09 | None | 4 + 5 = 9 | 5 | 62.00 | No | No | Yes | No | pT3a | pNx | M0 |

Abbreviations: NA, not available; None: No significant documented comorbidities were present. The prefix p indicates pathologic staging based on the pathological section; Nx denotes no regional lymph nodes retrieved for pathological examination; and M is determined by imaging results.

| **Table S4: Baseline Characteristics of Patients Grouped by the *In Vitro* Flutamide Metabolizing Capacity of Their Fecal Samples** | | | | | | | | | | | | | | |
| --- | --- | --- | --- | --- | --- | --- | --- | --- | --- | --- | --- | --- | --- | --- |
| Variables | | Total (n = 12) | | | | High Metabolizers  (HMP, ≥90%, n = 5) | | Low Metabolizers  (LMP, <90%, n = 7) | | | Statistic | *P* | | |
|  |  |  |  |  |  |  |  |  |  |  |  |  |  |  |
| Age, Mean ± SD | | 68.00 ± 7.59 | | | | 68.40 ± 5.94 | | 67.71 ± 9.05 | | | t = 0.15 | 0.886 | | |
| BMI (kg/m^2^),  M (Q₁, Q₃) | | 25.09 (23.21, 28.22) | | | | 23.66 (22.86, 28.51) | | 25.10 (24.21, 27.43) | | | Z = - 0.16 | 0.876 | | |
| PSA Level (ng/mL),  M (Q₁, Q₃) | | 10.00 (7.35, 38.55) | | | | 21.90 (9.00, 38.85) | | 9.61 (6.47, 27.49) | | | Z = - 0.46 | 0.648 | | |
| Family history, n (%) | |  | | | |  | |  | | | - | 1.000 | | |
| No | | 10 (83.33) | | | | 4 (80.00) | | 6 (85.71) | | |  |  | | |
| Yes | | 2 (16.67) | | | | 1 (20.00) | | 1 (14.29) | | |  |  | | |
| Smoking, n (%) | |  | | | |  | |  | | | - | 0.558 | | |
| No | | 7 (58.33) | | | | 2 (40.00) | | 5 (71.43) | | |  |  | | |
| Yes | | 5 (41.67) | | | | 3 (60.00) | | 2 (28.57) | | |  |  | | |
| Drinking, n (%) | |  | | | |  | |  | | | - | 0.293 | | |
| No | | 5 (41.67) | | | | 1 (20.00) | | 4 (57.14) | | |  |  | | |
| Yes | | 7 (58.33) | | | | 4 (80.00) | | 3 (42.86) | | |  |  | | |
| Gleason Grade Group, n (%) | |  | | | |  | |  | | | - | 0.740 | | |
| 1 | | 1 (8.34) | | | | 1 (20.00) | | 0 (0.00) | | |  |  | | |
| 2 | | 4 (33.34) | | | | 2 (40.00) | | 2 (28.57) | | |  |  | | |
| 3 | | 3 (25.00) | | | | 1 (20.00) | | 2 (28.57) | | |  |  | | |
| 4 | | 3 (25.00) | | | | 1 (20.00) | | 2 (28.57) | | |  |  | | |
| 5 | | 1 (8.34) | | | | 0 (0.00) | | 1 (14.29) | | |  |  | | |
| Abbreviations: t: t-test; Z: Mann-Whitney test; -: Fisher exact; SD: standard deviation, M: Median, Q₁: 1st Quartile, Q₃: 3rd Quartile | | | | | | | | | | | | | | |
| HMP: High-metabolizer population (*in vitro* Flutamide metabolism ≥90%); LMP: Low-metabolizer population (*in vitro* Flutamide metabolism <90%) | | | | | | | | | | | | | | |
| **Table S5: Mass Spectrometric Characterization of FLU-6 and FLU-9 by LC-MS (****Q-Exactive Orbitrap)** | | | | | | | | | | | | |  |  |
| Metabolites | Retention time  (min) | | Reaction | Molecular weight | Molecular formula | | ppm | | Fragment characteristics | | | | |  |
|  |  |  |  |  |  |  |  |  | Mass spectrometry (MS; [M−H] −) | MS/MS ([M−H] −) | | | |  |
| FLU-6 | 4.11 | | nitroreduction | 246.0980 | C_11_H_13_F_3_N_2_O | | 5.93 | | 245.09 | 225.08,205.08,185.07,175.05, 155.04,135.04,115.03 | | | |  |
| FLU-9 | 3.91 | | acetylation | 288.1086 | C_13_H_15_F_3_N_2_O_2_ | | 4.98 | | 287.10 | 245.09,225.08,205.08,185.07 | | | |  |

Note: LC-MS analysis was performed using a Q-Exactive Orbitrap mass spectrometer. All MS and MS/MS data were acquired in the negative ion mode.

| **Table S6: Homologs of *E. coli* NfsB Enzyme Identified by BLAST Search in Microbial Genomes.** | | | | | | |
| --- | --- | --- | --- | --- | --- | --- |
| Description | Genus | Identity (%) | E value | Score | Query cover (%) |  |
| *Escherichia coli* str. K-12 substr. MG1655 | *Escherichia* | 100.000 | 2.67E-160 | 448 | 100 |  |
| *Escherichia coli* UMN026 | *Escherichia* | 99.539 | 9.41E-160 | 447 | 100 |  |
| *Escherichia coli* O104:H4 str. 2011C-3493 | *Escherichia* | 99.078 | 5.82E-159 | 444 | 100 |  |
| *Shigella boydii* Sb227 | *Shigella* | 99.078 | 5.82E-159 | 444 | 100 |  |
| *Shigella sonnei* strain H140920393 | *Shigella* | 99.078 | 5.82E-159 | 444 | 100 |  |
| *Escherichia coli* O157:H7 str. Sakai | *Escherichia* | 99.078 | 5.82E-159 | 444 | 100 |  |
| *Shigella sonnei* Ss046 | *Shigella* | 99.078 | 5.82E-159 | 444 | 100 |  |
| *Escherichia coli* IAI39 | *Escherichia* | 99.078 | 6.57E-159 | 444 | 100 |  |
| *Shigella* sp. D9 | *Shigella* | 98.618 | 1.82E-158 | 443 | 100 |  |
| *Escherichia coli* O83:H1 str. NRG 857C | *Escherichia* | 98.618 | 9.45E-158 | 441 | 100 |  |
| *Shigella dysenteriae* Sd197 | *Shigella* | 98.618 | 2.13E-157 | 441 | 100 |  |
| *Escherichia fergusonii* ATCC 35469 | *Escherichia* | 97.696 | 1.87E-156 | 438 | 100 |  |
| *Escherichia albertii* TW07627 | *Escherichia* | 97.235 | 6.67E-156 | 437 | 100 |  |
| *Citrobacter koseri* ATCC BAA-895 | *Citrobacter* | 88.940 | 4.99E-144 | 407 | 100 |  |
| *Citrobacter rodentium* ICC168 | *Citrobacter* | 89.862 | 2.73E-142 | 402 | 100 |  |
| *Salmonella enterica* subsp. enterica serovar  Typhi str. Ty2 | *Salmonella* | 88.018 | 1.31E-141 | 401 | 100 |  |
| *Salmonella enterica* subsp. enterica serovar  Typhi str. CT18 | *Salmonella* | 88.018 | 1.31E-141 | 401 | 100 |  |
| *Salmonella enterica* subsp. enterica serovar  Typhimurium str. LT2 | *Salmonella* | 88.479 | 1.84E-141 | 400 | 100 |  |
| *Salmonella enterica* subsp. enterica serovar  Typhimurium str. 14028S | *Salmonella* | 88.479 | 1.84E-141 | 400 | 100 |  |

(**Table S6_continued)**

| *Salmonella enterica* subsp. enterica serovar *Paratyphi* A str. ATCC 9150 | *Salmonella* | 88.479 | 1.84E-141 | 400 | 100 |
| --- | --- | --- | --- | --- | --- |
| *Enterobacter lignolyticus* SCF1 | *Enterobacter* | 88.018 | 2.40E-141 | 400 | 100 |
| *Enterobacter asburiae* LF7a | *Enterobacter* | 88.018 | 1.13E-140 | 398 | 100 |
| *Enterobacter cancerogenus* ATCC 35316 | *Enterobacter* | 88.479 | 2.48E-140 | 397 | 100 |
| *Salmonella enterica* subsp. arizonae serovar  62:z4,z23:- strain RSK2980 | *Salmonella* | 87.097 | 1.15E-139 | 396 | 100 |
| *Citrobacter youngae* ATCC 29220 | *Citrobacter* | 87.097 | 1.65E-139 | 395 | 100 |
| *Leclercia adecarboxylata*  strain USDA-ARS-USMARC-60222 | *Leclercia* | 88.018 | 1.75E-139 | 395 | 100 |
| *Leclercia adecarboxylata* ATCC 23216 | *Leclercia* | 88.018 | 1.75E-139 | 395 | 100 |
| *Enterobacter mori* LMG 25706 | *Enterobacter* | 88.018 | 1.77E-139 | 395 | 100 |
| *Enterobacteriaceae bacterium* strain FGI 57 | *Enterobacter* | 87.558 | 2.62E-139 | 395 | 100 |
| *Raoultella ornithinolytica* B6 | *Raoultella* | 85.253 | 5.23E-139 | 394 | 100 |
| *Enterobacter cloacae* subsp. cloacae ATCC 13047 | *Enterobacter* | 87.558 | 1.27E-138 | 393 | 100 |
| *Enterobacter hormaechei* ATCC 49162 | *Enterobacter* | 87.097 | 3.85E-138 | 392 | 100 |
| *Citrobacter* sp. 30_2 | *Citrobacter* | 85.253 | 2.30E-137 | 390 | 100 |
| *Salmonella bongori* NCTC 12419 | *Salmonella* | 86.175 | 4.59E-137 | 389 | 100 |
| *Klebsiella* sp*.* RIT-PI-d | *Klebsiella* | 85.253 | 4.69E-137 | 389 | 100 |
| *Citrobacter freundii* CFNIH1 | *Citrobacter* | 85.714 | 6.89E-137 | 389 | 100 |
| *Escherichia vulneris* NBRC 102420 | *Escherichia* | 84.332 | 6.97E-137 | 389 | 100 |

(**Table S6_continued)**

| *Klebsiella pneumoniae* subsp. pneumoniae DSM 30104 | *Klebsiella* | 84.332 | 1.59E-135 | 385 | 100 |
| --- | --- | --- | --- | --- | --- |
| *Klebsiella oxytoca* strain CAV1374 | *Klebsiella* | 84.793 | 2.43E-136 | 387 | 100 |
| *Klebsiella variicola* At-22 | *Klebsiella* | 84.332 | 3.27E-136 | 387 | 100 |
| *Enterobacter aerogenes* KCTC 2190 | *Klebsiella* | 84.332 | 1.33E-135 | 385 | 100 |
| *Trabulsiella odontotermitis* strain TbO2.3 | *Trabulsiella* | 83.871 | 4.50E-135 | 384 | 100 |
| *Klebsiella pneumoniae* subsp*.* pneumoniae MGH 78578 | *Klebsiella* | 83.871 | 4.86E-135 | 384 | 100 |
| *Klebsiella oxytoca* KCTC 1686 | *Klebsiella* | 83.871 | 5.02E-135 | 384 | 100 |
| *Yokenella regensburgei* ATCC 43003 | *Yokenella* | 83.871 | 1.27E-134 | 383 | 100 |
| *Klebsiella pneumoniae* subsp*.* pneumoniae HS11286 | *Klebsiella* | 83.871 | 1.95E-134 | 382 | 100 |
| *Pluralibacter gergoviae* FB2 | *Pluralibacter* | 83.871 | 5.19E-134 | 382 | 100 |
| *Enterobacter* sp*.* 638 | *Enterobacter* | 84.793 | 5.54E-134 | 381 | 100 |
| *Kluyvera cryocrescens* NBRC 102467 | *Kluyvera* | 82.488 | 1.07E-132 | 378 | 100 |
| *Kluyvera ascorbata* ATCC 33433 | *Kluyvera* | 81.567 | 3.70E-131 | 374 | 100 |

Note: Query cover (%), Percentage of the query sequence covered by the alignment; Identity (%), Percentage of identical amino acid residues or nucleotides in the aligned region; E value, A lower value denotes greater confidence in the match.

| **Table S7: Homologs of *E. coli* NfsA Enzyme Identified by BLAST Search in Microbial Genomes.** | | | | | | |
| --- | --- | --- | --- | --- | --- | --- |
| Description | Genus | Query cover (%) | Identity (%) | E value | score |  |
| *Escherichia coli* str. K-12 substr. MG1655 | *Escherichia* | 100.00 | 100.000 | 2.34E-179 | 498 |  |
| *Shigella* sp. D9 | *Escherichia* | 100.00 | 100.000 | 2.34E-179 | 498 |  |
| *Escherichia coli* O104:H4 str. 2011C-3493 | *Escherichia* | 100.00 | 99.167 | 3.12E-177 | 493 |  |
| *Shigella dysenteriae* Sd197 | *Shigella* | 100.00 | 98.750 | 4.34E-177 | 492 |  |
| *Shigella boydii* Sb227 | *Shigella* | 100.00 | 98.750 | 3.37E-176 | 490 |  |
| *Shigella sonnei* strain H140920393 | *Shigella* | 100.00 | 98.750 | 3.37E-176 | 490 |  |
| *Shigella sonnei* Ss046 | *Shigella* | 100.00 | 98.750 | 3.37E-176 | 490 |  |
| *Escherichia coli* O157:H7 str. Sakai | *Escherichia* | 100.00 | 98.333 | 1.45E-176 | 491 |  |
| *Escherichia coli* O83:H1 str. NRG 857C | *Escherichia* | 100.00 | 98.333 | 7.19E-176 | 489 |  |
| *Shigella flexneri* 2a str. 301 | *Shigella* | 90.00 | 98.148 | 8.62E-155 | 436 |  |
| *Escherichia coli* UMN026 | *Escherichia* | 100.00 | 97.917 | 5.01E-176 | 489 |  |
| *Escherichia coli* IAI39 | *Escherichia* | 100.00 | 97.917 | 1.14E-175 | 489 |  |
| *Escherichia albertii* TW07627 | *Escherichia* | 87.92 | 95.261 | 7.03E-147 | 415 |  |
| *Escherichia fergusonii* ATCC 35469 | *Escherichia* | 100.00 | 91.250 | 2.61E-164 | 460 |  |
| *Enterobacter cloacae* subsp. cloacae ATCC 13047 | *Enterobacter* | 100.00 | 88.333 | 5.07E-153 | 431 |  |
| *Salmonella enterica* subsp. enterica serovar  Typhimurium str. LT2 | *Salmonella* | 100.00 | 87.917 | 3.61E-149 | 422 |  |
| *Salmonella enterica* subsp. enterica serovar  Typhimurium str. 14028S | *Salmonella* | 100.00 | 87.917 | 3.61E-149 | 422 |  |
| *Salmonella enterica* subsp. enterica serovar *Paratyphi* A str. ATCC 9150 | *Salmonella* | 100.00 | 87.917 | 1.08E-148 | 421 |  |
| *Citrobacter freundii* CFNIH1 | *Citrobacter* | 100.00 | 87.500 | 1.16E-159 | 448 |  |

(**Table S7_continued)**

| *Salmonella enterica* subsp. enterica serovar  Typhi str. Ty2 | *Salmonella* | 100.00 | 87.500 | 1.85E-148 | 420 |
| --- | --- | --- | --- | --- | --- |
| *Salmonella enterica* subsp. enterica serovar  Typhi str. CT18 | *Salmonella* | 100.00 | 87.500 | 1.85E-148 | 420 |
| *Salmonella enterica* subsp*.* arizonae  serovar 62:z4,z23:- strain RSK2980 | *Salmonella* | 100.00 | 87.500 | 4.26E-148 | 419 |
| *Salmonella bongori* NCTC 12419 | *Salmonella* | 100.00 | 87.500 | 7.62E-148 | 418 |
| *Enterobacter cancerogenus* ATCC 35316 | *Enterobacter* | 100.00 | 86.667 | 4.81E-149 | 421 |
| *Citrobacter* sp. 30_2 | *Citrobacter* | 100.00 | 86.250 | 1.71E-157 | 443 |
| *Citrobacter rodentium* ICC168 | *Citrobacter* | 100.00 | 86.250 | 6.46E-149 | 421 |
| *Citrobacter koseri* ATCC BAA-895 | *Citrobacter* | 100.00 | 86.250 | 1.19E-146 | 416 |
| *Enterobacter hormaechei* ATCC 49162 | *Enterobacter* | 100.00 | 85.833 | 2.52E-148 | 420 |
| *Enterobacter pulveris* DSM 19144 | *Enterobacter* | 100.00 | 85.833 | 6.39E-143 | 406 |
| *Citrobacter youngae* ATCC 29220 | *Citrobacter* | 100.00 | 85.417 | 1.39E-155 | 438 |
| *Enterobacter asburiae* LF7a | *Enterobacter* | 100.00 | 85.417 | 2.26E-147 | 417 |
| *Enterobacter mori* LMG 25706 | *Enterobacter* | 100.00 | 85.417 | 2.57E-147 | 417 |
| *Enterobacteriaceae bacterium* LSJC7 | *Enterobacter* | 100.00 | 85.000 | 1.60E-154 | 435 |
| *Enterobacteriaceae bacterium* strain FGI 57 | *Enterobacter* | 100.00 | 85.000 | 2.28E-140 | 399 |
| *Enterobacter* sp. 638 | *Enterobacter* | 100.00 | 84.167 | 1.05E-154 | 436 |
| *Enterobacter aerogenes* KCTC 2190 | *Enterobacter* | 100.00 | 84.167 | 3.24E-152 | 429 |
| *Enterobacter lignolyticus* SCF1 | *Enterobacter* | 100.00 | 84.167 | 2.13E-138 | 394 |

(**Table S7_continued)**

| *Klebsiella pneumoniae* subsp. pneumoniae  DSM 30104 | *Klebsiella* | 100.00 | 83.750 | 1.05E-146 | 416 |
| --- | --- | --- | --- | --- | --- |
| *Leclercia adecarboxylata*  strain USDA-ARS-USMARC-60222 | *Leclercia* | 100.00 | 83.750 | 3.00E-145 | 412 |
| *Leclercia adecarboxylata* ATCC 23216 | *Leclercia* | 100.00 | 83.750 | 3.00E-145 | 412 |
| *Escherichia hermannii* NBRC 105704 | *Escherichia* | 100.00 | 83.333 | 2.66E-146 | 414 |
| *Klebsiella pneumoniae* subsp*.* pneumoniae  MGH 78578 | *Klebsiella* | 100.00 | 83.333 | 7.21E-146 | 413 |
| *Klebsiella variicola* At-22 | *Klebsiella* | 100.00 | 83.333 | 7.45E-146 | 413 |
| *Yokenella regensburgei* ATCC 43003 | *Yokenella* | 100.00 | 82.917 | 8.41E-151 | 426 |
| *Kosakonia sacchari* SP1 | *Kosakonia* | 100.00 | 82.917 | 5.78E-139 | 396 |
| *Enterobacter cloacae* subsp. dissolvens SP1 | *Enterobacter* | 100.00 | 82.917 | 5.78E-139 | 396 |
| *Cronobacter sakazakii* ATCC BAA-894 | *Cronobacter* | 100.00 | 82.083 | 1.77E-141 | 402 |
| *Cronobacter sakazakii* strain ATCC 29544 | *Cronobacter* | 100.00 | 82.083 | 1.77E-141 | 402 |
| *Cronobacter malonaticus* 681 | *Cronobacter* | 100.00 | 82.083 | 4.80E-141 | 401 |
| *Kluyvera intermedia* strain CAV1151 | *Kluyvera* | 100.00 | 82.083 | 1.91E-139 | 397 |
| *Cronobacter universalis* NCTC 9529 | *Cronobacter* | 100.00 | 81.667 | 1.66E-140 | 400 |
| *Raoultella ornithinolytica* B6 | *Raoultella* | 100.00 | 81.667 | 2.20E-140 | 399 |
| *Kluyvera cryocrescens* NBRC 102467 | *Kluyvera* | 100.00 | 81.667 | 3.06E-140 | 399 |

(**Table S7_continued)**

| *Cronobacter turicensis* z3032 | *Cronobacter* | 100.00 | 81.250 | 1.81E-140 | 400 |
| --- | --- | --- | --- | --- | --- |
| *Cronobacter zurichensis* LMG 23730 | *Cronobacter* | 100.00 | 81.250 | 8.68E-140 | 398 |
| *Kluyvera ascorbata* ATCC 33433 | *Kluyvera* | 100.00 | 81.250 | 1.84E-134 | 388 |
| *Klebsiella oxytoca* strain CAV1374 | *Kluyvera* | 100.00 | 80.833 | 7.68E-138 | 393 |
| *Buttiauxella agrestis* strain MCE | *Buttiauxella* | 100.00 | 80.417 | 2.43E-139 | 397 |
| *Klebsiella oxytoca* KCTC 1686 | *Klebsiella* | 100.00 | 80.417 | 2.04E-137 | 392 |
| *Cedecea neteri* SSMD04 | *Cedecea* | 100.00 | 80.417 | 7.50E-135 | 385 |
| *Cedecea neteri* M006 | *Cedecea* | 100.00 | 80.000 | 7.19E-137 | 390 |
| *Klebsiella* sp. RIT-PI-d | *Klebsiella* | 100.00 | 80.000 | 3.62E-132 | 379 |
| *Trabulsiella odontotermitis* strain TbO2.3 | *Trabulsiella* | 100.00 | 80.000 | 2.88E-131 | 377 |

Note: Query cover (%), Percentage of the query sequence covered by the alignment; Identity (%), Percentage of identical amino acid residues or nucleotides in the aligned region; E value, A lower value denotes greater confidence in the match.

| **Table S8: Mass Spectrometric Characterization of FLU-6 and FLU-5 by LC-MS (Orbitrap Exploris^TM^ 120)** | | | | | | | |
| --- | --- | --- | --- | --- | --- | --- | --- |
| Metabolites | Retention time (min) | Reaction | Molecular weight | Molecular formula | ppm | Fragment characteristics | |
|  |  |  |  |  |  | Mass spectrometry (MS; [M−H] −) | MS/MS ([M−H] −) |
| FLU-6 | 3.244 | nitroreduction | 246.0980 | C_11_H_13_F_3_N_2_O | 0.12 | 245.09 | 225.08,205.08,185.07,175.05, 155.04,135.04,115.03 |
| FLU-5 | 2.996 | hydroxylation | 262.0929 | C_13_H_15_F_3_N_2_O_2_ | 1.65 | 261.09 | 175.05,155.04,135.04,115.03 |

Note: LC-MS analysis was performed using a Orbitrap Exploris^TM^ 120 mass spectrometer. All MS and MS/MS data were acquired in the negative ion mode.

| **Table S9: ADMET Prediction for Flutamide Metabolites FLU-6 and FLU-9** | | |
| --- | --- | --- |
| Metabolite | Property | Value^1^ |
| FLU-6 | OATP1B3 inhibitor | ++ |
| FLU-9 |  | ++ |
| FLU-6 | MRP1 inhibitor | ++ |
| FLU-9 |  | +++ |
| FLU-6 | Pgp-inhibitor | ++ |
| FLU-9 |  | + |

Note: ADMET (Absorption, Distribution, Metabolism, Excretion, Toxicity) properties were predicted in silico using the ADMETlab 3.0 platform. The results focus on the potential drug-drug interaction risk via key drug transporters.

^1^ The plus sign system indicates the predicted inhibition strength on the corresponding transporter, based on the ADMETlab 3.0 model output: + (Weak inhibition); ++ (Moderate inhibition); +++ (Strong inhibition).

| **Table S10: Interindividual Variability in Flutamide Metabolism and FLU-6 Formation by Human Gut Microbiota** | | | |
| --- | --- | --- | --- |
| No. | Flutamide Metabolized (%)^1^ | FLU-6 peak area (6h, mean) |  |
| P_01 | 91.31 | 3.42E+09 |  |
| P_02 | 91.86 | 2.96E+09 |  |
| P_03 | 53.69 | 1.13E+09 |  |
| P_04 | 59.21 | 5.55E+08 |  |
| P_05 | 91.07 | 2.07E+09 |  |
| P_06 | 6.14 | 3.51E+08 |  |
| P_07 | 57.38 | 1.72E+09 |  |
| P_08 | 99.58 | 2.92E+09 |  |
| P_09 | 93.17 | 2.26E+09 |  |
| P_10 | 28.02 | 1.56E+09 |  |
| P_11 | 35.81 | 1.75E+09 |  |
| P_12 | 55.00 | 1.24E+08 |  |

^1^ Flutamide Metabolized (%) = (Flutamide Peak Area_0h_ -Flutamide Peak Area_6h_)/Flutamide Peak Area_0h_*100%

| **Table S11.** **Relative Abundance of Major Gut Microbial Species in the Study Cohort.** ^1^ | | | | | | | | | | | | |  |
| --- | --- | --- | --- | --- | --- | --- | --- | --- | --- | --- | --- | --- | --- |
| Species | P_01 | P_05 | P_08 | P_02 | P_09 | P_12 | P_06 | P_10 | P_04 | P_11 | P_07 | P_03 | average |
| *Segatella_copri* | 0.0868 | 0.0044 | 0.0024 | 0.0040 | 0.1378 | 0.0037 | 0.3991 | 0.0119 | 0.0434 | 0.2238 | 0.4014 | 0.0021 | 0.1101 |
| *Bacteroides*_unclassified^2^ | 0.1605 | 0.1497 | 0.0995 | 0.1811 | 0.0824 | 0.0443 | 0.0045 | 0.0646 | 0.0779 | 0.0758 | 0.0107 | 0.0092 | 0.0800 |
| *Phocaeicola_vulgatus* | 0.0300 | 0.0379 | 0.0910 | 0.1603 | 0.0219 | 0.1970 | 0.0047 | 0.0361 | 0.0202 | 0.0303 | 0.0122 | 0.0701 | 0.0593 |
| *Prevotellaceae*_unclassified^2^ | 0.0442 | 0.0021 | 0.0010 | 0.0015 | 0.0999 | 0.0017 | 0.1905 | 0.0055 | 0.0203 | 0.1064 | 0.2077 | 0.0009 | 0.0568 |
| *Escherichia_coli* | 0.0030 | 0.0172 | 0.3469 | 0.0204 | 0.0056 | 0.0029 | 0.0026 | 0.0028 | 0.0106 | 0.0025 | 0.0003 | 0.0155 | 0.0359 |
| *Bacteroidales*_unclassified^2^ | 0.0434 | 0.0562 | 0.0187 | 0.0605 | 0.0487 | 0.0193 | 0.0183 | 0.0368 | 0.0437 | 0.0527 | 0.0156 | 0.0089 | 0.0352 |
| *Phocaeicola*_unclassified^2^ | 0.0106 | 0.0185 | 0.0381 | 0.0681 | 0.0083 | 0.0851 | 0.0014 | 0.0145 | 0.0084 | 0.0115 | 0.0045 | 0.0291 | 0.0248 |
| *Bacteroides_uniformis* | 0.0814 | 0.0694 | 0.0057 | 0.0272 | 0.0191 | 0.0095 | 0.0029 | 0.0257 | 0.0337 | 0.0080 | 0.0044 | 0.0024 | 0.0241 |
| *Phocaeicola_plebeius* | 0.0270 | 0.0117 | 0.0011 | 0.0028 | 0.0012 | 0.0021 | 0.0871 | 0.0833 | 0.0029 | 0.0024 | 0.0133 | 0.0017 | 0.0197 |
| *Phocaeicola_dorei* | 0.0061 | 0.0581 | 0.0222 | 0.0328 | 0.0068 | 0.0379 | 0.0023 | 0.0113 | 0.0184 | 0.0129 | 0.0039 | 0.0133 | 0.0188 |
| *Bacteroidaceae*_unclassified^2^ | 0.0183 | 0.0284 | 0.0167 | 0.0444 | 0.0102 | 0.0387 | 0.0060 | 0.0171 | 0.0151 | 0.0137 | 0.0046 | 0.0119 | 0.0188 |
| *Alistipes*_unclassified^2^ | 0.0077 | 0.0668 | 0.0002 | 0.0016 | 0.0226 | 0.0003 | 0.0002 | 0.0324 | 0.0518 | 0.0227 | 0.0122 | 0.0014 | 0.0183 |
| *Bacteroides_ovatus* | 0.0165 | 0.0137 | 0.0283 | 0.0577 | 0.0224 | 0.0154 | 0.0017 | 0.0078 | 0.0053 | 0.0314 | 0.0042 | 0.0016 | 0.0172 |
| *Faecalibacterium_prausnitzii* | 0.0096 | 0.0029 | 0.0004 | 0.0008 | 0.0202 | 0.0461 | 0.0007 | 0.0099 | 0.0083 | 0.0159 | 0.0093 | 0.0562 | 0.0150 |
| *Bacteroides_stercoris* | 0.0124 | 0.0638 | 0.0014 | 0.0034 | 0.0172 | 0.0019 | 0.0013 | 0.0055 | 0.0418 | 0.0017 | 0.0048 | 0.0138 | 0.0141 |
| *[Eubacterium]_rectale* | 0.0007 | 0.0003 | 0.0000 | 0.0029 | 0.0021 | 0.0020 | 0.0003 | 0.0017 | 0.0109 | 0.0089 | 0.0010 | 0.1216 | 0.0127 |
| *Bacteria*_unclassified^2^ | 0.0123 | 0.0112 | 0.0111 | 0.0213 | 0.0101 | 0.0141 | 0.0097 | 0.0136 | 0.0118 | 0.0144 | 0.0091 | 0.0089 | 0.0123 |
| *Bacteroides_thetaiotaomicron* | 0.0047 | 0.0073 | 0.0209 | 0.0287 | 0.0204 | 0.0104 | 0.0076 | 0.0076 | 0.0089 | 0.0148 | 0.0059 | 0.0013 | 0.0116 |
| *Roseburia_inulinivorans* | 0.0007 | 0.0018 | 0.0000 | 0.0001 | 0.0009 | 0.0019 | 0.0003 | 0.0040 | 0.0020 | 0.0006 | 0.0017 | 0.1209 | 0.0112 |
| *Bacteroides_fragilis* | 0.0054 | 0.0094 | 0.0148 | 0.0419 | 0.0075 | 0.0214 | 0.0038 | 0.0052 | 0.0049 | 0.0138 | 0.0025 | 0.0012 | 0.0110 |
| *Eubacteriales*_unclassified^2^ | 0.0112 | 0.0086 | 0.0020 | 0.0011 | 0.0100 | 0.0117 | 0.0009 | 0.0158 | 0.0102 | 0.0097 | 0.0119 | 0.0378 | 0.0109 |
| *Roseburia_intestinalis* | 0.0054 | 0.0018 | 0.0000 | 0.0002 | 0.0056 | 0.1064 | 0.0001 | 0.0012 | 0.0008 | 0.0008 | 0.0020 | 0.0037 | 0.0107 |
| *Phocaeicola_coprocola* | 0.0970 | 0.0030 | 0.0018 | 0.0036 | 0.0012 | 0.0017 | 0.0022 | 0.0033 | 0.0060 | 0.0018 | 0.0047 | 0.0009 | 0.0106 |
| *Enterobacteriaceae*_unclassified^2^ | 0.0006 | 0.0044 | 0.0857 | 0.0052 | 0.0013 | 0.0010 | 0.0005 | 0.0006 | 0.0036 | 0.0005 | 0.0001 | 0.0047 | 0.0090 |

(**Table S11_continued)**

| *Bacteroides_xylanisolvens* | 0.0054 | 0.0086 | 0.0146 | 0.0203 | 0.0116 | 0.0056 | 0.0021 | 0.0040 | 0.0042 | 0.0137 | 0.0021 | 0.0012 | 0.0078 |
| --- | --- | --- | --- | --- | --- | --- | --- | --- | --- | --- | --- | --- | --- |
| *Parabacteroides*_unclassified^2^ | 0.0033 | 0.0071 | 0.0016 | 0.0273 | 0.0083 | 0.0025 | 0.0003 | 0.0071 | 0.0115 | 0.0157 | 0.0027 | 0.0010 | 0.0073 |
| *Alistipes_putredinis* | 0.0024 | 0.0233 | 0.0002 | 0.0013 | 0.0082 | 0.0003 | 0.0002 | 0.0097 | 0.0250 | 0.0084 | 0.0035 | 0.0008 | 0.0069 |
| *Parabacteroides_distasonis* | 0.0032 | 0.0072 | 0.0027 | 0.0231 | 0.0057 | 0.0014 | 0.0006 | 0.0056 | 0.0080 | 0.0162 | 0.0027 | 0.0013 | 0.0065 |
| *Bacteroides_caccae* | 0.0097 | 0.0130 | 0.0048 | 0.0035 | 0.0051 | 0.0013 | 0.0004 | 0.0077 | 0.0156 | 0.0111 | 0.0021 | 0.0016 | 0.0063 |
| *Megamonas*_unclassified^2^ | 0.0044 | 0.0000 | 0.0001 | 0.0001 | 0.0000 | 0.0001 | 0.0652 | 0.0018 | 0.0013 | 0.0000 | 0.0001 | 0.0003 | 0.0061 |
| *Faecalibacterium*_unclassified^2^ | 0.0039 | 0.0009 | 0.0001 | 0.0001 | 0.0057 | 0.0171 | 0.0002 | 0.0032 | 0.0033 | 0.0063 | 0.0044 | 0.0218 | 0.0056 |
| *Parabacteroides_merdae* | 0.0046 | 0.0085 | 0.0010 | 0.0071 | 0.0066 | 0.0032 | 0.0009 | 0.0078 | 0.0128 | 0.0073 | 0.0022 | 0.0013 | 0.0053 |
| *Prevotella_stercorea* | 0.0008 | 0.0002 | 0.0001 | 0.0002 | 0.0088 | 0.0001 | 0.0297 | 0.0012 | 0.0114 | 0.0018 | 0.0040 | 0.0001 | 0.0049 |
| *Roseburia*_unclassified^2^ | 0.0010 | 0.0013 | 0.0000 | 0.0001 | 0.0009 | 0.0140 | 0.0001 | 0.0013 | 0.0031 | 0.0011 | 0.0059 | 0.0285 | 0.0048 |
| *Megamonas_funiformis* | 0.0031 | 0.0001 | 0.0000 | 0.0001 | 0.0000 | 0.0001 | 0.0482 | 0.0013 | 0.0013 | 0.0000 | 0.0001 | 0.0003 | 0.0046 |
| *Odoribacter_splanchnicus* | 0.0011 | 0.0074 | 0.0002 | 0.0005 | 0.0018 | 0.0004 | 0.0003 | 0.0112 | 0.0241 | 0.0006 | 0.0029 | 0.0003 | 0.0042 |
| *Flavonifractor_plautii* | 0.0026 | 0.0274 | 0.0011 | 0.0015 | 0.0041 | 0.0020 | 0.0007 | 0.0025 | 0.0026 | 0.0035 | 0.0009 | 0.0008 | 0.0042 |
| *Phocaeicola_massiliensis* | 0.0006 | 0.0010 | 0.0011 | 0.0016 | 0.0321 | 0.0017 | 0.0005 | 0.0077 | 0.0005 | 0.0006 | 0.0004 | 0.0008 | 0.0040 |
| *Clostridium*_unclassified^2^ | 0.0088 | 0.0013 | 0.0003 | 0.0003 | 0.0063 | 0.0005 | 0.0010 | 0.0082 | 0.0026 | 0.0054 | 0.0020 | 0.0116 | 0.0040 |
| *[Ruminococcus]_gnavus* | 0.0005 | 0.0014 | 0.0049 | 0.0018 | 0.0007 | 0.0036 | 0.0064 | 0.0009 | 0.0007 | 0.0008 | 0.0003 | 0.0247 | 0.0039 |
| *Phascolarctobacterium_faecium* | 0.0113 | 0.0097 | 0.0001 | 0.0001 | 0.0119 | 0.0001 | 0.0002 | 0.0003 | 0.0005 | 0.0086 | 0.0001 | 0.0011 | 0.0037 |
| *Oscillospiraceae_bacterium* | 0.0011 | 0.0014 | 0.0000 | 0.0000 | 0.0088 | 0.0007 | 0.0002 | 0.0092 | 0.0037 | 0.0012 | 0.0017 | 0.0157 | 0.0036 |
| *Bacillota*_unclassified^2^ | 0.0022 | 0.0029 | 0.0003 | 0.0003 | 0.0055 | 0.0043 | 0.0011 | 0.0047 | 0.0041 | 0.0024 | 0.0018 | 0.0106 | 0.0034 |
| *Prevotella*_unclassified^2^ | 0.0002 | 0.0001 | 0.0000 | 0.0000 | 0.0165 | 0.0000 | 0.0077 | 0.0003 | 0.0107 | 0.0008 | 0.0022 | 0.0001 | 0.0032 |
| *Bacteroides_finegoldii* | 0.0019 | 0.0024 | 0.0032 | 0.0168 | 0.0020 | 0.0024 | 0.0003 | 0.0047 | 0.0017 | 0.0020 | 0.0005 | 0.0004 | 0.0032 |
| *Lachnospira_eligens* | 0.0004 | 0.0012 | 0.0001 | 0.0001 | 0.0028 | 0.0013 | 0.0001 | 0.0133 | 0.0005 | 0.0055 | 0.0091 | 0.0022 | 0.0030 |
| *[Eubacterium]_siraeum* | 0.0001 | 0.0001 | 0.0000 | 0.0000 | 0.0003 | 0.0004 | 0.0001 | 0.0330 | 0.0003 | 0.0003 | 0.0006 | 0.0008 | 0.0030 |

(**Table S11_continued)**

| *Hscolarctobacterium*_unclassified^2^ | 0.0084 | 0.0077 | 0.0001 | 0.0000 | 0.0110 | 0.0000 | 0.0001 | 0.0006 | 0.0003 | 0.0062 | 0.0001 | 0.0009 | 0.0030 |
| --- | --- | --- | --- | --- | --- | --- | --- | --- | --- | --- | --- | --- | --- |
| *Butyrivibrio*_sp._CAG:318 | 0.0000 | 0.0000 | 0.0000 | 0.0000 | 0.0000 | 0.0001 | 0.0001 | 0.0340 | 0.0001 | 0.0002 | 0.0000 | 0.0001 | 0.0029 |
| *Bacteroides_intestinalis* | 0.0009 | 0.0015 | 0.0008 | 0.0012 | 0.0019 | 0.0005 | 0.0003 | 0.0211 | 0.0037 | 0.0011 | 0.0003 | 0.0002 | 0.0028 |
| *Oscillospiraceae*_unclassified^2^ | 0.0030 | 0.0134 | 0.0001 | 0.0000 | 0.0026 | 0.0003 | 0.0001 | 0.0027 | 0.0056 | 0.0020 | 0.0014 | 0.0010 | 0.0027 |
| *Haemophilus_parainfluenzae* | 0.0000 | 0.0000 | 0.0000 | 0.0000 | 0.0000 | 0.0287 | 0.0000 | 0.0016 | 0.0000 | 0.0003 | 0.0001 | 0.0001 | 0.0026 |
| *Alistipes_onderdonkii* | 0.0037 | 0.0119 | 0.0002 | 0.0003 | 0.0012 | 0.0002 | 0.0001 | 0.0019 | 0.0040 | 0.0034 | 0.0037 | 0.0002 | 0.0026 |
| *Lachnospiraceae*_unclassified^2^ | 0.0022 | 0.0011 | 0.0004 | 0.0003 | 0.0013 | 0.0042 | 0.0006 | 0.0019 | 0.0023 | 0.0019 | 0.0019 | 0.0120 | 0.0025 |
| *Alistipes_*sp*.*_CAG:268 | 0.0000 | 0.0001 | 0.0000 | 0.0000 | 0.0001 | 0.0001 | 0.0001 | 0.0237 | 0.0002 | 0.0002 | 0.0055 | 0.0001 | 0.0025 |
| *Bacteroides_cellulosilyticus* | 0.0009 | 0.0012 | 0.0027 | 0.0010 | 0.0095 | 0.0004 | 0.0002 | 0.0035 | 0.0050 | 0.0044 | 0.0002 | 0.0002 | 0.0024 |
| *Roseburia_hominis* | 0.0046 | 0.0002 | 0.0000 | 0.0001 | 0.0003 | 0.0012 | 0.0002 | 0.0021 | 0.0083 | 0.0023 | 0.0053 | 0.0045 | 0.0024 |
| *Enterocloster_bolteae* | 0.0034 | 0.0041 | 0.0047 | 0.0030 | 0.0008 | 0.0014 | 0.0006 | 0.0005 | 0.0052 | 0.0023 | 0.0003 | 0.0013 | 0.0023 |
| *Bilophila_wadsworthia* | 0.0028 | 0.0097 | 0.0002 | 0.0014 | 0.0000 | 0.0002 | 0.0003 | 0.0007 | 0.0085 | 0.0032 | 0.0003 | 0.0001 | 0.0023 |
| *Blautia*_unclassified^2^ | 0.0006 | 0.0010 | 0.0000 | 0.0001 | 0.0011 | 0.0024 | 0.0001 | 0.0006 | 0.0009 | 0.0007 | 0.0004 | 0.0189 | 0.0022 |
| *Prevotella_pectinovora* | 0.0008 | 0.0001 | 0.0000 | 0.0000 | 0.0215 | 0.0000 | 0.0004 | 0.0002 | 0.0026 | 0.0003 | 0.0004 | 0.0001 | 0.0022 |
| *Klebsiella_pneumoniae* | 0.0003 | 0.0015 | 0.0159 | 0.0004 | 0.0001 | 0.0023 | 0.0001 | 0.0002 | 0.0023 | 0.0004 | 0.0001 | 0.0011 | 0.0021 |
| *Phocaeicola_coprophilus* | 0.0147 | 0.0007 | 0.0003 | 0.0003 | 0.0007 | 0.0003 | 0.0010 | 0.0011 | 0.0008 | 0.0005 | 0.0013 | 0.0001 | 0.0018 |
| *Eubacterium_ventriosum* | 0.0023 | 0.0004 | 0.0001 | 0.0001 | 0.0004 | 0.0009 | 0.0001 | 0.0010 | 0.0055 | 0.0027 | 0.0009 | 0.0069 | 0.0018 |
| *uncultured_bacterium* | 0.0009 | 0.0008 | 0.0011 | 0.0013 | 0.0014 | 0.0007 | 0.0052 | 0.0016 | 0.0012 | 0.0014 | 0.0012 | 0.0023 | 0.0016 |
| *Odoribacter*_unclassified^2^ | 0.0000 | 0.0027 | 0.0000 | 0.0000 | 0.0005 | 0.0000 | 0.0000 | 0.0044 | 0.0099 | 0.0000 | 0.0011 | 0.0001 | 0.0016 |
| *Clostridiales_bacterium* | 0.0009 | 0.0006 | 0.0000 | 0.0001 | 0.0026 | 0.0001 | 0.0000 | 0.0081 | 0.0042 | 0.0007 | 0.0011 | 0.0002 | 0.0016 |
| *Bacteroides_nordii* | 0.0004 | 0.0029 | 0.0015 | 0.0026 | 0.0014 | 0.0005 | 0.0001 | 0.0022 | 0.0032 | 0.0030 | 0.0003 | 0.0001 | 0.0015 |

(**Table S11_continued)**

| *Fusobacterium*_unclassified^2^ | 0.0002 | 0.0002 | 0.0004 | 0.0165 | 0.0000 | 0.0002 | 0.0003 | 0.0000 | 0.0000 | 0.0001 | 0.0001 | 0.0000 | 0.0015 |
| --- | --- | --- | --- | --- | --- | --- | --- | --- | --- | --- | --- | --- | --- |
| *Butyricimonas_virosa* | 0.0018 | 0.0005 | 0.0001 | 0.0005 | 0.0061 | 0.0001 | 0.0001 | 0.0011 | 0.0021 | 0.0026 | 0.0029 | 0.0000 | 0.0015 |
| *Enterocloster*_unclassified^2^ | 0.0021 | 0.0025 | 0.0040 | 0.0021 | 0.0003 | 0.0007 | 0.0004 | 0.0001 | 0.0034 | 0.0014 | 0.0001 | 0.0003 | 0.0014 |
| *Roseburia_faecis* | 0.0004 | 0.0012 | 0.0000 | 0.0001 | 0.0003 | 0.0008 | 0.0000 | 0.0008 | 0.0038 | 0.0014 | 0.0064 | 0.0021 | 0.0014 |
| *Akkermansia_muciniphila* | 0.0001 | 0.0003 | 0.0016 | 0.0000 | 0.0001 | 0.0003 | 0.0000 | 0.0008 | 0.0001 | 0.0138 | 0.0001 | 0.0001 | 0.0014 |
| *Dialister_succinatiphilus* | 0.0001 | 0.0000 | 0.0000 | 0.0000 | 0.0000 | 0.0000 | 0.0000 | 0.0001 | 0.0167 | 0.0000 | 0.0001 | 0.0001 | 0.0014 |
| *Alistipes_shahii* | 0.0006 | 0.0037 | 0.0000 | 0.0001 | 0.0026 | 0.0001 | 0.0001 | 0.0049 | 0.0016 | 0.0025 | 0.0004 | 0.0001 | 0.0014 |
| *Blautia_obeum* | 0.0008 | 0.0006 | 0.0003 | 0.0003 | 0.0007 | 0.0013 | 0.0003 | 0.0008 | 0.0008 | 0.0006 | 0.0005 | 0.0096 | 0.0014 |
| *Streptococcus*_unclassified^2^ | 0.0000 | 0.0000 | 0.0000 | 0.0001 | 0.0001 | 0.0152 | 0.0001 | 0.0006 | 0.0001 | 0.0001 | 0.0001 | 0.0001 | 0.0014 |
| *Butyricimonas*_unclassified^2^ | 0.0014 | 0.0003 | 0.0002 | 0.0006 | 0.0055 | 0.0002 | 0.0003 | 0.0015 | 0.0016 | 0.0023 | 0.0023 | 0.0001 | 0.0014 |
| *Blautia_wexlerae* | 0.0005 | 0.0009 | 0.0001 | 0.0001 | 0.0012 | 0.0024 | 0.0001 | 0.0005 | 0.0007 | 0.0006 | 0.0003 | 0.0087 | 0.0013 |
| *Ruthenibacterium_lactatiformans* | 0.0003 | 0.0072 | 0.0005 | 0.0000 | 0.0020 | 0.0002 | 0.0000 | 0.0017 | 0.0018 | 0.0006 | 0.0004 | 0.0005 | 0.0013 |
| *Prevotella_copri*_CAG:164 | 0.0011 | 0.0000 | 0.0000 | 0.0000 | 0.0005 | 0.0000 | 0.0053 | 0.0002 | 0.0006 | 0.0030 | 0.0044 | 0.0000 | 0.0013 |
| *Akkermansia*_unclassified | 0.0000 | 0.0000 | 0.0033 | 0.0000 | 0.0000 | 0.0002 | 0.0000 | 0.0013 | 0.0000 | 0.0103 | 0.0000 | 0.0000 | 0.0013 |
| *Oscillibacter*_sp._ER4 | 0.0001 | 0.0001 | 0.0000 | 0.0000 | 0.0043 | 0.0000 | 0.0000 | 0.0008 | 0.0078 | 0.0001 | 0.0013 | 0.0003 | 0.0012 |
| *Ruminococcus*_unclassified^2^ | 0.0002 | 0.0018 | 0.0000 | 0.0000 | 0.0001 | 0.0001 | 0.0000 | 0.0016 | 0.0026 | 0.0001 | 0.0005 | 0.0075 | 0.0012 |
| *Prev*otella_sp._AM34-19LB | 0.0004 | 0.0001 | 0.0000 | 0.0000 | 0.0060 | 0.0000 | 0.0014 | 0.0000 | 0.0003 | 0.0011 | 0.0051 | 0.0000 | 0.0012 |
| *Lachnospiraceae_bacterium* | 0.0020 | 0.0009 | 0.0000 | 0.0001 | 0.0007 | 0.0001 | 0.0001 | 0.0008 | 0.0007 | 0.0005 | 0.0003 | 0.0082 | 0.0012 |
| *Haemophilus*_unclassified^2^ | 0.0000 | 0.0000 | 0.0000 | 0.0000 | 0.0000 | 0.0131 | 0.0000 | 0.0007 | 0.0000 | 0.0001 | 0.0001 | 0.0001 | 0.0012 |
| *Streptococcus_salivarius* | 0.0000 | 0.0000 | 0.0000 | 0.0002 | 0.0001 | 0.0128 | 0.0001 | 0.0005 | 0.0001 | 0.0001 | 0.0001 | 0.0000 | 0.0012 |

(**Table S11_continued)**

| *Firmicutes_bacterium*_CAG:124 | 0.0001 | 0.0000 | 0.0000 | 0.0000 | 0.0004 | 0.0000 | 0.0000 | 0.0099 | 0.0034 | 0.0001 | 0.0000 | 0.0001 | 0.0012 |
| --- | --- | --- | --- | --- | --- | --- | --- | --- | --- | --- | --- | --- | --- |
| *Dorea_longicatena* | 0.0009 | 0.0007 | 0.0001 | 0.0002 | 0.0009 | 0.0004 | 0.0001 | 0.0006 | 0.0015 | 0.0004 | 0.0004 | 0.0076 | 0.0011 |
| *Veillonella*_unclassified^2^ | 0.0000 | 0.0000 | 0.0051 | 0.0001 | 0.0000 | 0.0078 | 0.0001 | 0.0001 | 0.0000 | 0.0001 | 0.0001 | 0.0002 | 0.0011 |
| *Bacteroides_*sp._D20 | 0.0056 | 0.0032 | 0.0002 | 0.0010 | 0.0007 | 0.0003 | 0.0000 | 0.0009 | 0.0013 | 0.0002 | 0.0001 | 0.0001 | 0.0011 |
| *Bacteroides_faecis* | 0.0014 | 0.0036 | 0.0006 | 0.0020 | 0.0010 | 0.0010 | 0.0002 | 0.0012 | 0.0007 | 0.0012 | 0.0002 | 0.0001 | 0.0011 |
| *Dysosmobacter_segnis* | 0.0001 | 0.0002 | 0.0000 | 0.0000 | 0.0015 | 0.0001 | 0.0000 | 0.0028 | 0.0044 | 0.0020 | 0.0016 | 0.0002 | 0.0011 |
| *Sutterella*_sp._AM11-39 | 0.0000 | 0.0017 | 0.0000 | 0.0000 | 0.0000 | 0.0000 | 0.0014 | 0.0011 | 0.0000 | 0.0063 | 0.0019 | 0.0003 | 0.0011 |
| *Prevotella*_sp._CAG:617 | 0.0000 | 0.0001 | 0.0000 | 0.0000 | 0.0001 | 0.0000 | 0.0001 | 0.0000 | 0.0000 | 0.0000 | 0.0122 | 0.0000 | 0.0011 |
| *Paraprevotella_clara* | 0.0064 | 0.0004 | 0.0001 | 0.0003 | 0.0002 | 0.0002 | 0.0005 | 0.0007 | 0.0005 | 0.0006 | 0.0024 | 0.0000 | 0.0010 |
| *Ruminococcaceae_bacterium*_TF06-43 | 0.0001 | 0.0001 | 0.0000 | 0.0000 | 0.0033 | 0.0000 | 0.0000 | 0.0010 | 0.0061 | 0.0001 | 0.0012 | 0.0004 | 0.0010 |
| *Alistipes_indistinctus* | 0.0000 | 0.0001 | 0.0002 | 0.0001 | 0.0043 | 0.0000 | 0.0001 | 0.0003 | 0.0057 | 0.0012 | 0.0004 | 0.0000 | 0.0010 |
| *Alistipes_finegoldii* | 0.0003 | 0.0021 | 0.0001 | 0.0001 | 0.0008 | 0.0001 | 0.0000 | 0.0025 | 0.0048 | 0.0008 | 0.0006 | 0.0001 | 0.0010 |
| Cumulative Abundance of Remaining Taxa^3^ | 0.1536 | 0.1311 | 0.1085 | 0.0851 | 0.1606 | 0.1583 | 0.0636 | 0.2814 | 0.2425 | 0.1181 | 0.1270 | 0.2357 | 0.1555 |

^1^ The data represents the relative abundance of each microbial species based on shotgun metagenomic sequencing and normalized to 1.

^2^ Taxa labeled as "unclassified" could not be definitively identified to that taxonomic level.

^3^ Cumulative Abundance of Remaining Taxa represents the aggregated relative abundance of all microbial sequences excluded from the table due to their mean relative abundance being below the 0.001 inclusion threshold.

**Abbreviations:** P_01 to P_12, Patient identification numbers (corresponding to Table S3).
