# Supplementary material for: Gut microbial metabolism of Flutamide attenuates its therapeutic efficacy against prostate cancer
Source: Gut Microbes. 2026 Jun 7;18(1):2682803. doi: 10.1080/19490976.2026.2682803 (PMC13248909; doi:10.1080/19490976.2026.2682803)
Supplement: Author Affiliation Change Agreement.pdf [file KGMI_A_2682803_SM2940.pdf]

Author Affiliation Change Agreement

Manuscript Title: Gut Microbial Metabolism of Flutamide Attenuates Its Therapeutic Efficacy Against Prostate Cancer

Manuscript Number: 245076945

We, the undersigned authors, hereby confirm our unanimous agreement to correct the order of the institutional affiliations, as follows:

Original affiliation:

1. College of Pharmaceutical Science, Zhejiang University of Technology
2. Department of Pharmacy, Zhejiang Cancer Hospital, Hangzhou Institute of Medicine (HIM), Chinese Academy of Sciences

Corrected affiliations:

1. Department of Pharmacy, Zhejiang Cancer Hospital, Hangzhou Institute of Medicine (HIM), Chinese Academy of Sciences
2. College of Pharmaceutical Science, Zhejiang University of Technology

We understand and confirm that this correction concerns only the order of institutional affiliations. It does not affect the order of authors, author contributions, or author qualifications.

Signatures of all authors:

1. **Shujing Li (First Author)**

   Signature: Shujing Li Date: 4 December 2025

2. **Haiying Ding (Co-First Author)**

   Signature: Haiying Ding Date: 4 December 2025

3. **Jiaqi Wang (Co-First Author)**

   Signature: Jiaqi Wang Date: 4 December 2025

4. **Lingjie Yuan (Author)**

   Signature: Lingjie Yuan Date: 4 December 2025

5. **Ying Zhou (Author)**

Signature: Ying zhou Date: 4 December 2025

6. **Weiben Xu (Author)**

Signature: Weiben Xu Date: 4 December 2025

7. **Hang Yin (Author)**

Signature: Hang Yin Date: 4 December 2025

8. **Mengqian Ye (Author)**

Signature: Mengqian Ye Date: 4 December 2025

9. **Yuning Sha (Author)**

Signature: Yuning sha Date: 4 December 2025

10. **Fangyin Li (Author)**

Signature: FangYin Li Date: 4 December 2025

11. **Yousheng Liu (Author)**

Signature: Yousheng Liu Date: 4 December 2025

12. **Zhengqin Zhu (Author)**

Signature: Zhengqin Zhu Date: 4 December 2025

13. **Lulu Song (Author)**

Signature: Lulu Song Date: 4 December 2025

14. **Xiangyu Jin (Author)**

Signature: Xiangyu Jin Date: 4 December 2025

15. **LieFeng Ma (Author)**

Signature: LieFeng Ma Date: 4 December 2025

16. **Zhajun Zhan (Corresponding Author)**

Signature: Zhajun zhan Date: 4 December 2025

17. **Libin Pan (Corresponding Author)**

Signature: Libin Pan Date: 4 December 2025

18. **Luo Fang (Corresponding Author, Lead contact)**

Signature: luo fang Date: 4 December 2025
